# Differential transcriptome analysis reveals genes related to cold tolerance in seabuckthorn carpenter moth, *Eogystia hippophaecolus*

**DOI:** 10.1371/journal.pone.0187105

**Published:** 2017-11-13

**Authors:** Mingming Cui, Ping Hu, Tao Wang, Jing Tao, Shixiang Zong

**Affiliations:** 1 Beijing Key Laboratory for Forest Pest Control, Beijing Forestry University, Beijing, P.R. China; 2 Mentougou Forestry Station, Beijing, China; USDA Agricultural Research Service, UNITED STATES

## Abstract

Seabuckthorn carpenter moth, *Eogystia hippophaecolus* (Lepidoptera: Cossidae), is an important pest of sea buckthorn (*Hippophae rhamnoides*), which is a shrub that has significant ecological and economic value in China. *E*. *hippophaecolus* is highly cold tolerant, but limited studies have been conducted to elucidate the molecular mechanisms underlying its cold resistance. Here we sequenced the *E*. *hippophaecolus* transcriptome using RNA-Seq technology and performed *de novo* assembly from the short paired-end reads. We investigated the larval response to cold stress by comparing gene expression profiles between treatments. We obtained 118,034 unigenes, of which 22,161 were annotated with gene descriptions, conserved domains, gene ontology terms, and metabolic pathways. These resulted in 57 GO terms and 193 Kyoto Encyclopedia of Genes and Genomes (KEGG) pathways. By comparing transcriptome profiles for differential gene expression, we identified many differentially expressed proteins and genes, including heat shock proteins and cuticular proteins which have previously been reported to be involved in cold resistance of insects. This study provides a global transcriptome analysis and an assessment of differential gene expression in *E*. *hippophaecolus* under cold stress. We found seven differential expressed genes in common between developmental stages, which were verified with qPCR. Our findings facilitate future genomic studies aimed at improving our understanding of the molecular mechanisms underlying the response of insects to low temperatures.

## Introduction

Insects are poikilothermic animals and thus temperature is one of the most important abiotic factors influencing their distribution and behavior [1,2]. Nearly every aspect of an insect’s life is affected by temperature, including survival, development, reproduction, and longevity [3–6]. Nevertheless, insects have evolved to flourish in a wide variety of thermal environments [3]. They have achieved this through the natural selection of a variety of strategies to cope with changes in ambient temperatures. Some insects overwinter by migrating to warmer climes or by moving to protected habitats [7–9]. Most insects, however, survive through the winter as a result of a variety of physiological and biochemical adaptations that can be grouped into two general strategies: freeze intolerance or freeze tolerance [10–12]. Freeze-intolerant insects are very sensitive to ice formation. They avoid freezing by supercooling to survive at low temperatures [1]. They achieve this by reducing intra-cellular water content, synthesizing cryoprotectants, and actively removing ice-nucleating agents (INAs) [7,12,13]. Compared with freeze intolerance, the freeze-tolerant strategy is more advanced because it permits extracellular ice formation within tissues. Ice formation is triggered by INAs such as insect ice nucleating proteins (INPs), which are often lipoproteins [14, 15].

To date, considerable research has been conducted on the biochemical mechanisms of insect cold resistance. Increasingly, genomic and proteomic screening methods are being applied to discover the genes and proteins that enable winter survival, which is greatly broadening our understanding of important molecular processes. Two key proteins have so far been recognized to directly contribute to insect cold hardiness: anti-freeze proteins (AFPs) and INPs [16]. Other proteins contribute to cold hardiness indirectly, including heat-shock proteins (HSPs), antioxidant enzymes, and enzymes involved in cryoprotectant synthesis and degradation [17]. In the transcriptomic studies of Dunning et al., (2013) and Dennis et al., (2015), a set of structural cuticular genes were identified to be associated with stick insect adaptation to the alpine environment. [18, 19]. They also identified three candidate genes that responded to low temperature: prolyl 4-hydroxylase subunit alpha-1, staphylococcal nuclease domain-containing protein 1 and a cuticular protein gene [20]. Durant et al’s (2015) research on transcription profiling of the brain in *Ceratina calcarata* showed that changes in gene expression associated with overwintering, included an underrepresentation of genes related to muscle fibers and an overrepresentation of genes related to lipid metabolic processes [21].

Because RNA transcript levels generally reflect physiological demand, changes in transcript abundance should identify genes involved in an organism’s response to extrinsic stimuli [22]. Transcriptome analysis using Next Generation technologies has been proven to be a powerful method for exploring the underlying basis of the cold response in a variety of insect lineages. Examples include the study of transcriptional profiling of overwintering gene expression in a carpenter bee [21], the identification of cold-responsive genes in an alpine stick insect [20], the analysis of divergent transcriptional responses in alpine and lowland stick insects [18], and the study of cold adaptation in eight genera stick insects [19]. Compared with traditional differential genes expression methods such as expressed sequence tags (EST), microarrays, serial analysis of gene expression (SAGE), or massively parallel signature sequencing (MPSS), a complete transcriptome sequence and analysis can be completed for any species with high efficiency without a reference genome. Moreover, transcripts normally expressed at very low levels can be detected and quantified [23–26]. While transcriptome analysis have been used in studies of the cold response of *Drosophila melanogaster* and *D*. *subobscura* [27–29], its application to study cold hardiness in other insects has been quite limited.

The seabuckthorn carpenter moth *Eogystia hippophaecolus* (Hua, Chou, Fang & Chen, 1990) is the dominant boring insect pest of sea buckthorn (*Hippophae rhamnoides* L.). Sea buckthorn is a shrub that is distributed across northern and western China. The plant grows well in sandy soils in cold climates. It has been extensively planted to prevent soil erosion and desertification, and also to serve as a source for food and medicine products [30]. *E*. *hippophaecolus* completes a generation every three to four years and most of its life history is spent in 16 larval stages lasting about 31 months [31]. Eggs are mainly deposited in wounds or in cracks of the bark. The egg stage lasts 7–30 days. The early larvae feed on trunk phloem at first. Small larvae often congregate in large numbers and later migrate to the base of the truck or to the roots before winter. Larvae are very cold tolerant and can survive in temperatures lower than -30°C. The larvae obstruct water transport in sea buckthorn plants by burying into the trunk and roots. The shape of the galleries is irregular and most are not connected to each other. Each gallery is filled with sawdust and fecula. The mature larvae will climb out of the wormhole, and then burrow 15 cm deep into the soil in June. The pupal stage lasts about 30 days and is followed by eclosion, mating, egg-laying, and hatching in July, followed by overwintering in October [32]. Its voraciousness, high reproduction rate, and inconspicuous habit make the pest difficult to control. In China, seabuckthorn carpenter moth is considered a major threat to sea buckthorns, and it can also feed on other plants, such as elms [33,34]. In preliminary studies measuring physiological indices (e.g., SCP, freezing point, etc.) of cold resistance in *E*. *hippophaecolus*, we classified the seabuckthorn carpenter moth as a highly freeze-tolerant organism [35].

In this study, we used RNA-Seq and *de novo* transcriptome assembly to generate transcriptomes and examine the changes in the regulation of transcription associated with cold treatment. Detailed differential expression analysis revealed a number of candidate genes that are potentially related to the cold tolerance of *E*. *hippophaecolus*. Our results provide new genetic information about this insect pest and provide a rich resource for the discovery and identification of novel genes involved in the cold stress response.

## Materials and methods

### Insect material and RNA extraction

*E*. *hippophaecolus* larvae were collected from Jianping County, Chaoyang City, Liaoning Province, China, in late December 2014. The latitude and longitude of the collection site are 119.637° E and 41.3954° N. Given the long life history and many larval stages of this species, we chose three developmental stages (seventh instar, tenth instar, and thirteenth instar) [31] and two treatment temperatures to characterize variation in gene expression. Healthy larvae in different developmental stages were treated as presented in Table 1. Prior to treatment, we cleaned the moths with sterile water and starved them for three days. All of the instruments used to handle the moths were sterilized before use. After housing for 48 h at room temperature (25°C), three samples of each development stage were treated differently: one stayed at room temperature as a control and the other two were kept in 5°C and -5°C, respectively, for 10 h, and then snap-frozen in liquid nitrogen and stored at -80°C until the RNA extraction step. Total RNA was extracted using TRIzol reagent (Ambion) and the RNeasy Plus Mini Kit (No. 74134; Qiagen, Hilden, Germany) following the manufacturer’s instructions. A NanoDrop 8000 (Thermo, Waltham, MA, USA) was used to quantify the RNA. The mRNA samples were purified and fragmented using the TruSeq RNA Sample Preparation Kit v2-Set A (No. RS-122-2001; Illumina, San Diego, CA, USA) to remove rRNA. After additional quality control, cDNA library construction, Illumina sequencing, and *de novo* transcriptome assembly were performed by the Beijing Biomarker Biotechnology Co. (Beijing, China). In view of the costs, 5°C and -5°C samples were pooled by instar to construct the cDNA library. This method has been used in previous transcriptome studies [36, 37]. No specific permits were required for the described field studies. The location is not privately owned or protected, and the field studies did not involve endangered or protected species.

**Table 1:** Schematic table of the experimental design used to investigate effects of cold stress.

	control group	Treatment group	
Developmental stage	25°C	5°C	5°C
7th instar	T1	T4	
10th instar	T2	T5	
13th instar	T3	T6	

Note: There was one larva in each different treatment. After treatment, the total RNA of larvae under 5°C and 5°C were mixed for sequencing library construction. Samples were named T1~T6.

### Illumina sequencing and *de novo* transcript assembly

We sequenced the *E*. *hippophaecolus* transcriptome on an Illumina HiSeq 2500 platform and performed *de novo* assembly from the short paired-end reads (200bp). Before transcript assembly, all raw reads were processed to remove low-quality and adaptor sequences by Trimmomatic 0.32 (http://www.usadellab.org/cms/index.php?page=trimmomatic) using the following parameters (Phred64 LEADING:3 TRAILING:3 SLIDING WINDOW:4:15 MINLEN:36), while the unpaired reads were discarded [38]. The clean reads were then assembled into contigs and the contigs were subsequently combined into transcripts based on paired-end information using the Trinity platform (Version: r2013.11.10) [39]. CD-HIT was used to exclude redundant sequences and the clustering was implemented using TGICL (2.1) [40] to combine sequences with more than 90% similarity and an overlapping length longer than 35bp. The completeness assessment was performed by BUSCO (version 2), using the insecta lineage [41]. The transcripts were blasted to various RNA databases including Silva, GtRNAdb by BLAST software (version 2.2.26) for filtering (e-value≤10^−5^). After filtering, ribosomal RNA (rRNA), transfer RNA (tRNA), small nuclear RNA (snRNA), microRNA (miRNA), small nucleolar RNA (snoRNA), repeat sequences, and other non-coding RNA (ncRNA) were removed. The list of the sequences identified as ncRNA has been shown in S1 Table.

The raw data has been submitted to the NCBI Sequence Read Archive (SRA) with accession number SRR4409152. The mapped read counts have been submitted to the Gene Expression Omnibus (GEO) with accession number GSE92686.

### Gene functional annotation

We utilized BLAST software (version 2.2.26) [42] to obtain homologous sequences (e-value≤10^−5^) by searching several public protein databases, including the National Center for Biotechnology Information (NCBI) non-redundant (nr) protein database (downloaded on 02/2015) [43], the Swiss-Prot protein database 51.0 [44], the Gene Ontology (GO) database (downloaded on 12/2014) [45], the Kyoto Encyclopedia of Genes and Genomes (KEGG) pathway database 73.1 [46], and the Cluster of Orthologous Groups (COG) database (downloaded on 2013) [47]. We used the single best blast hit only to identify the contig and to extract GO information, while non-arthropod contigs were filtered out.

After predicting the amino-acid sequences of unique genes by using Transdecoder (downloaded on 2015), we obtained functional information about the unigenes by comparing these to the Pfam database using HMMER software (e-value≤10^−10^) [48].

### Differential gene expression analysis

Bowtie (4.4.7) [49] was used to align reads to reference sequences. Gene expression levels were measured in the RNA-Seq analysis as reads per kilobase of exon model per million mapped reads (RPKM) [50]. The EBSeq software was used to identify differentially expressed genes in pair-wise comparisons and separate models were fit for each development stage[51]. The results of all statistical tests were corrected for multiple testing with the Benjamini–Hochberg false discovery rate with the following parameter values: FDR < 0.01. Sequences were deemed to be significantly differentially expressed if the adjusted P value obtained by this method was <0.001 and there was at least a twofold change (>1 or <-1 in log2 ratio value which were calculated by the average RPKM value of treatment libraries divided by the average RPKM value of control libraries). K-Means clustering of gene expression was performed using the R Package software v.3.1.3 to examine the relationships between samples. GO annotation and KEGG pathway analysis were also performed to assess the differential expression results. To annotate the gene with gene ontology (GO) terms, the nr BLAST results were imported into the Blast2GO program (Conesa, Gotz et al. 2005). GO annotations for the genes were obtained by Blast2GO. This analysis mapped all of the annotated genes to GO terms in the database and counted the number of genes associated with each term. A Perl script was then used to plot GO functional classification for the unigenes with a GO term hit to view the distribution of gene functions. The gene sequences were also aligned to the Clusters of Orthologous Group (COG) database to predict and classify functions (Tatusov, Galperin et al. 2000). We used KOBAS (http://kobas.cbi.pku.edu.cn/home.do) to annotate a set of sequences with KO terms and identify both the most frequent and the statistically significantly enriched pathways. KEGG pathways were tested for enrichment using the right-sided Fisher's exact test. We removed the T3 transcript and its corresponding control, T6, from the DEG analysis because of large, unexplained variation or low correlation between the T3 and other samples.

#### Gene expression validation by real-time quantitative PCR

The seven genes DE in response to cold treatment in both developmental stages were chosen for validation using qRT-PCR. Total RNA was reverse transcribed using the PrimeScript RT Reagent Kit with gDNA Eraser (No. RR047A; TaKaRa, Shiga, Japan). Gene-specific primers were designed using Primer3 (http://bioinfo.ut.ee/primer3-0.4.0/). qRT-PCR was performed on a Bio-Rad CFX96 real-time PCR System (Hercules, CA, USA) using SYBR Premix Ex-Taq II (No. RR820A; TaKaRa), according to the manufacturer's instructions.

The template cDNAs were diluted 20-fold with nuclease-free deionized water. Of this, 2 μl was added to the reaction mixture (25 μl), which included 12.5 μl of SYBR Premix Ex Taq II, 1 μl of each primer (10 μM), and 8.5 μl of ddH_2_O. The PCR was performed with the following thermal cycle: 95°C for 30 s, 40 cycles at 95°C for 5 s, 60°C for 30 s and 65°C to 95°C in increments of 0.5°C for 5 s to generate melting curves. Each reaction was performed on three biological and four technical replicates. The average threshold cycle was calculated to estimate the relative gene expression levels using the 2^−ΔΔCt^ method [52]. The data were expressed as the mean ± SE, and all primer information is listed in S2 Table. A t-test was performed for the qPCR data. β-Actin was used as the reference gene to normalize target gene expression [53].

## Results

### Sequencing and assembly

A total of 39.00 Gb of clean data passed the Illumina quality filter after transcriptome sequencing of six cDNA samples. The number of the paired-end reads and the length of these reads are provided in S3 Table. The Q30 and GC contents were 88.30% and 44.93%, respectively. The quality control result of six samples was seen in S3 Table. The *de novo* assembly using Trinity software and standard parameter values yielded 191,200 transcripts (none shorter than 200 bp) with an N50 of 1,687 bp. The unigene dataset included 118,034 sequences with an N50 of 904bp. The length distribution of unigenes closely followed the length distribution of transcripts S1 Fig). Coverage of clean reads mapped to the assembly was provided in S3 Table. The completeness assessment result showed that 90.0% of BUSCO genes were “complete”, of which 84.3% were single-copy BUSCOs and 5.7% were duplicated BUSCOs. 6.2% were “fragmented”, while the remaining 3.8% were “missing”.

### Annotation of predicted proteins

After annotation, genes with a significant blast hit to arthropods were identified. In total, 22,161 unigenes were found in at least one public database, such as the nr database, the Swiss-Prot protein database, the GO database, the KEGG database, and the COG database. The SwissProt database (21,452 annotated unigenes, 21.47%) had the most matches, followed by the nr database (21,232, 21.25%). A total of 77,735 (77.82%) unigenes were not found in the public databases (Table 2). Overall, most of the unigenes either could not be annotated or their descriptions were uninformative (e.g., putative, unknown, hypothetical, or unnamed protein). Among the annotated unigenes, more than 70% of them matched with other lepidopteran species. The best homologous species match in the nr database for each sequence is shown in S2 Fig. The top matched species were the silkworm (*Bombyx mori*), with 8627 unigenes (40.73%), followed by the monarch butterfly (*Danaus plexippus*). The blast information for each contig was provided in S4 Table.

**Table 2:** Statistics of annotation analysis of unigenes.

Databases	Unigene	300≤length<1000	length≥1000
COG_Annotation	4,452	692	3,272
GO_Annotation	10,525	2,613	5,578
KEGG_Annotation	7,611	1,708	4,257
KOG_Annotation	10,784	2,266	6,763
Pfam_Annotation	12,413	2,893	7,857
Swissprot_Annotation	21,452	6,629	9,654
nr_Annotation	21,232	6,540	9,621
All	22,161	6,967	9,723

### Global functional classification of unigenes by GO and KEGG

**Fig 1:**
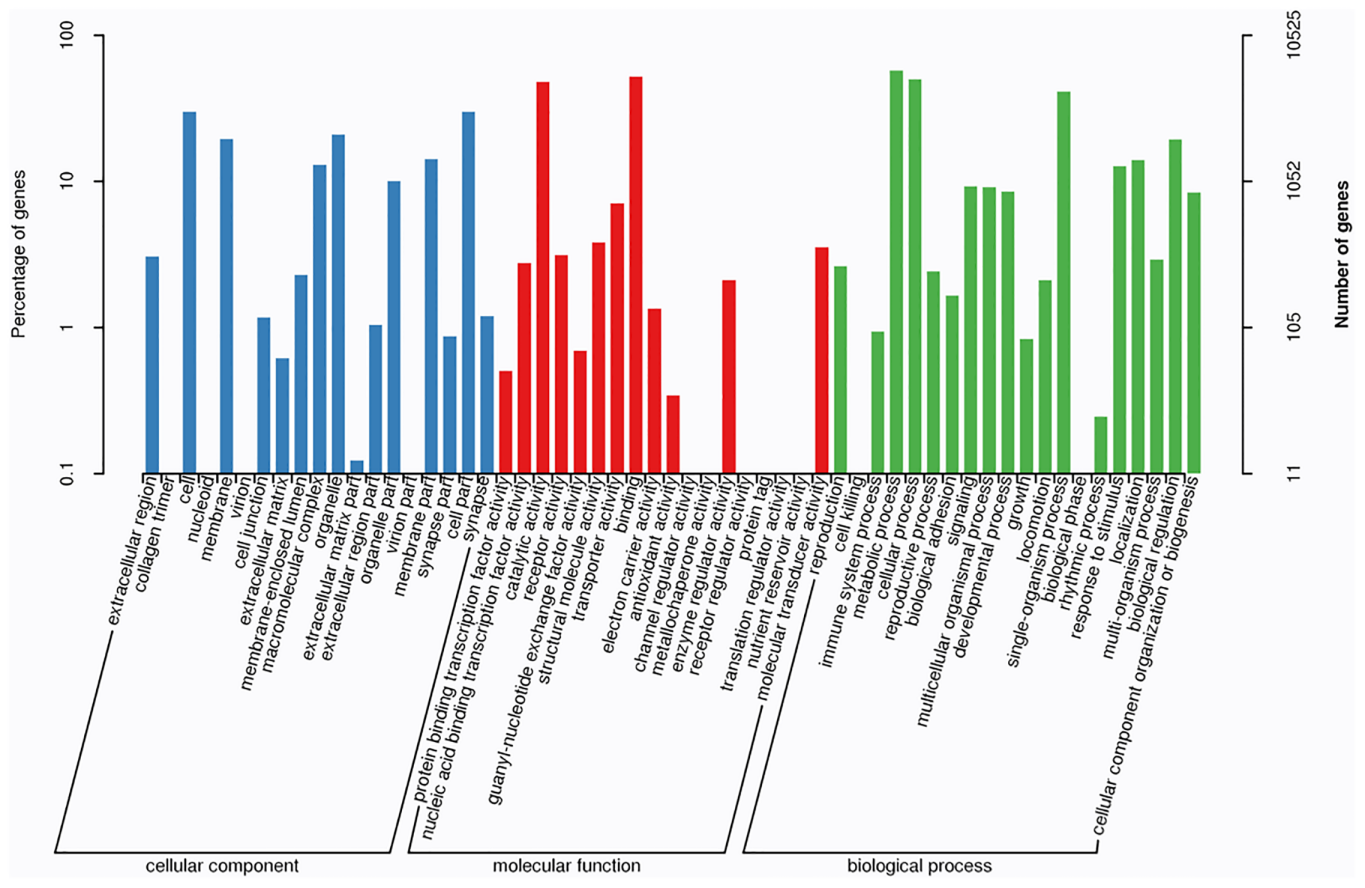
Gene ontology (GO) annotations of the annotated unigenes. 38,435 unigenes were assigned to three GO categories containing 57 functional subcategories. The y-axis on the right shows the number of genes in category, and that on the left shows the percentage of a specific category of genes in that main category.

One unigene can have different functional annotations; therefore, 38,435 unigenes in total with a match in the GO database were classified by their GO terms. The unigenes could be categorized into 57 functional sub-groups of the three main GO groups: 15,798 unigenes fell in the biological process group, 6,721 in the cellular component group, and 15,916 in the molecular function group (Fig 1). The most frequent GO terms were: metabolic process (6,071 unigenes), binding (5,492 unigenes), cellular process (5,258 unigenes), catalytic activity (5,067 unigenes), single-organism process (4,354 unigenes), cell part (3,159 unigenes), cell (3,156 unigenes).

**Fig 2:**
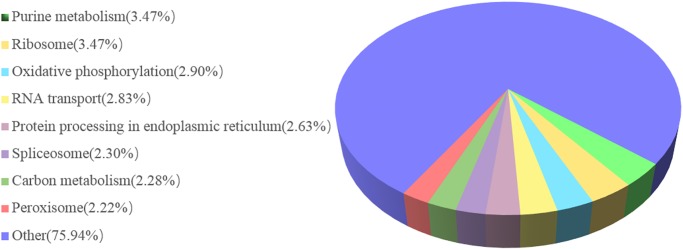
Pathway assignment based on KEGG mapping.

Pathway enrichment analysis based on the KEGG database was used to identify enriched metabolic pathways or signal transduction pathways. In total, 5,990 unigenes were assigned to 193 pathways (Fig 2). The pathway categories with the largest number of unigenes were purine metabolism and ribosome (both 208 unigenes), followed by oxidative phosphorylation (174), RNA transport (170), Protein processing in endoplasmic reticulum (158), spliceosome (138), carbon metabolism (137), peroxisome (133), and lysosome (115).

### DEGs in *E*. *hippophaecolus* larvae under cold stress

The correlations between samples were shown in S3 Fig. Based on the correlations, the clustering of samples was presented in S4 Fig. It can be seen sample T3 was not correlated well with other samples (r^2^ all less than 0.6). Use of such samples is likely to result in the spurious detection of DEG, and so sample T3 and its corresponding control, T6, were excluded from the DEG analysis. The remaining two groups (T1-T4, T2-T5) were then used to explore the genes involved in cold resistance. The DE information for each contig was provided in S5 Table.

**Fig 3:**
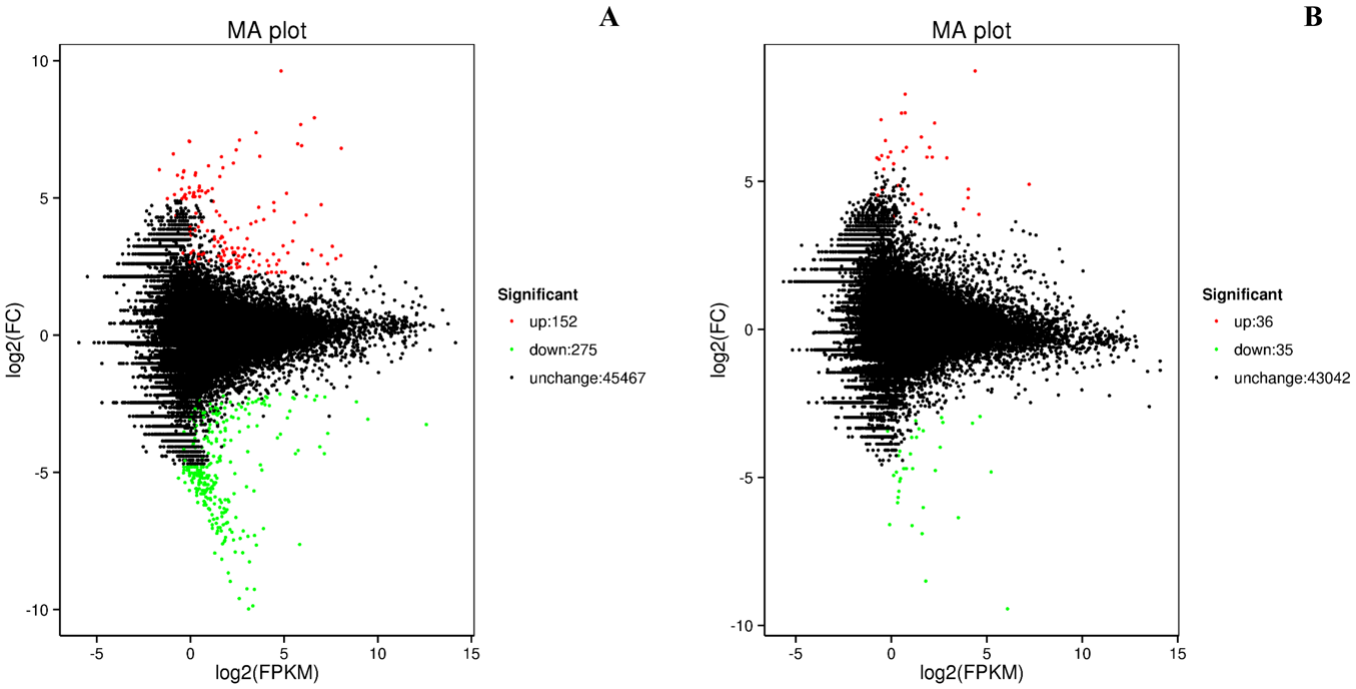
Distribution of DEGs between samples. (A) Distribution of DEGs between T1 and T4. (B) Distribution of DEGs between T2 and T5.

**Fig 4:**
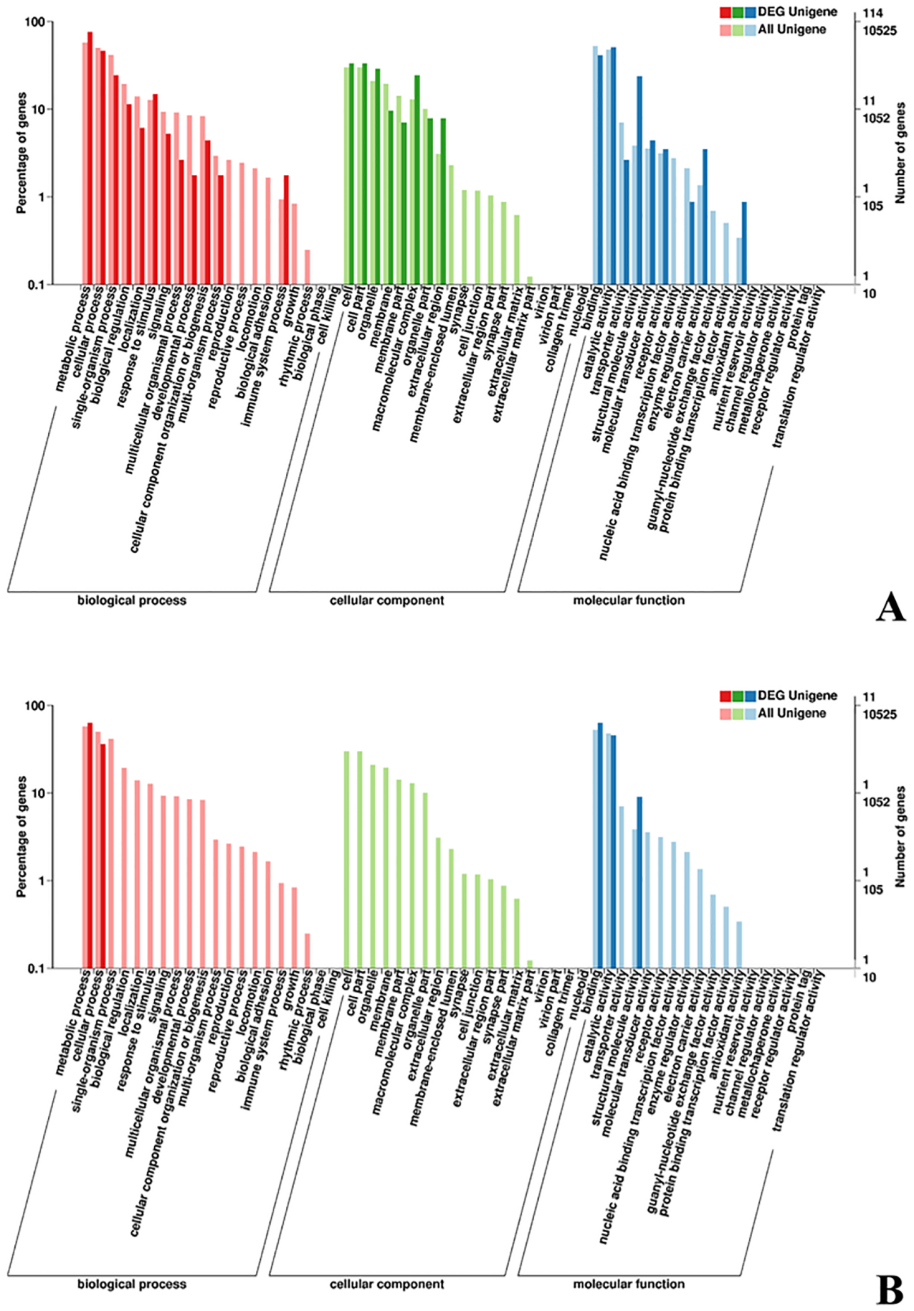
GO annotations of the DEGs. (A) GO annotations of the DEGs between T1 and T4. (B) GO annotations of the DEGs between T2 and T5.

We applied a rigorous procedure to identify DEGs (see Methods) and identified 427 unigenes differentially expressed between group T1 and T4, and 71 between group T2 and T5 (Fig 3). About half of the DEGs had annotations. The annotations of the DEGs were shown in S6 Table. Only seven DEGs were shared by the two groups, three of which were annotated. The blast results of these seven genes were shown in Table 3. GO annotation was used to evaluate the function of DEGs that were significantly different between treatments (Fig 4). Between T1 and T4, the most significant items were “Biological Process: metabolic process”, “Molecular Function: catalytic activity”, “Biological Process: cellular process”, “Molecular Function: binding”, and “Cellular Component: cell part” (Fig 4a). When comparing T2 and T5, unigenes were assigned to “Biological Process: metabolic process”, “Molecular Function: binding”, “Molecular Function: catalytic activity”, “Biological Process: cellular process” and “Molecular Function: structural molecule activity” (Fig 4b).

**Table 3:** Common DEGs differentially expressed genes in two groups (T1-T4 and T2-T5) from RNAseq.

Unigene ID	Blast Blastx Match				Log_2_FC^a^	Regulated^a^	Log_2_FC^b^	Regulated^b^
	annotation	Acc. Number	E-value	Identity				
c79869.graph_c1	endonuclease-reverse transcriptase [*Bombyx mori*]	ADI61830.1	0	64%	3.567980225	up	3.829149437	up
c76833.graph_c0	PREDICTED: uncharacterized protein LOC105570877 [*Vollenhovia emeryi*]	XP_011883719.1	1.00E-152	47%	3.587111514	down	4.534550285	up
c73192.graph_c0	PREDICTED: uncharacterized protein LOC106708429 isoform X1 [*Papilio machaon*]	XP_014355420.1	0	64%	6.026349523	up	7.944625711	up
c59923.graph_c0	putative axonemal leucine-rich repeat protein [*Danaus plexippus*]	EHJ70571.1	5.00E-128	67%	2.600939044	normal	2.969087192	normal
c33229.graph_c0	--	--	--	--	9.625223032	up	8.727798365	up
c36418.graph_c0	--	--	--	--	2.841664353	up	5.818570542	up
c68914.graph_c0	--	--	--	--	2.459464841	up	6.968321714	up

Note: FC^a^ indicates the ratio of RPKM of T4 sample divided by the RPKM of T1 sample; **FC**^**b**^ indicates the ratio of RPKM of T5 sample divided by the RPKM of T2 sample; Regulated^a^ indicates the expression level of DEGs in T4 compared to T1, including three situations, up-regulated, down-regulated or kept unchanged; other columns are functional annotations of unigenes; Regulated^b^ indicates the expression level of DEGs in T5 compared to T2; “--” means no information.

**Fig 5:**
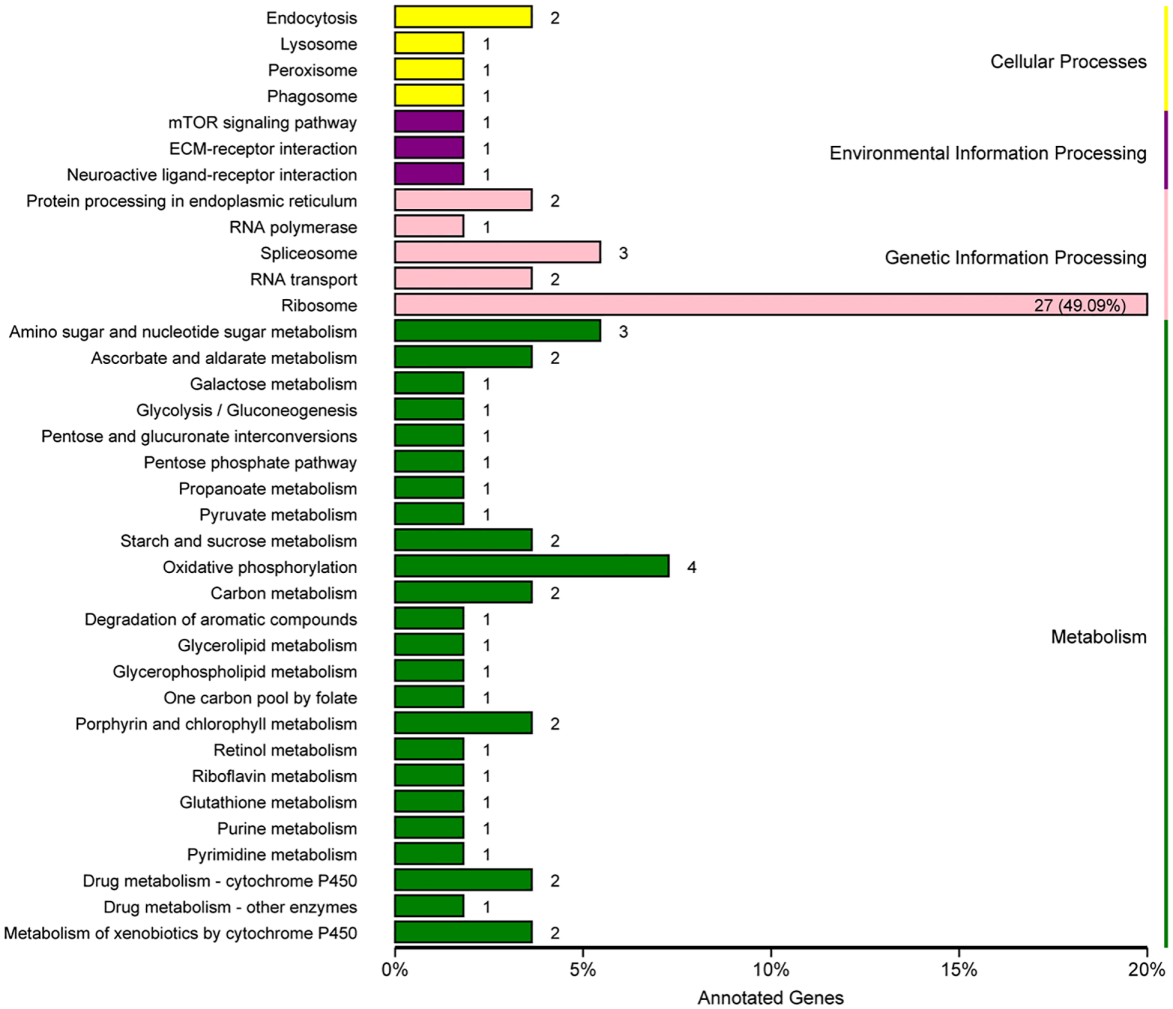
Pathways enriched for DEGs in T1-T4. The y-axis indicates the pathways and the x-axis indicates the percentage of unigenes in each pathway.

KEGG pathway enrichment analysis revealed the primary DEG pathways (Fig 5). Only group T1-T4 had pathway enrichment result, because DEGs between T2 and T5 were too few to be identified in the enrichment analysis. The most remarkable pathway was “ribosome”, which is dominated by ribosomal proteins. Another pathway was “oxidative phosphorylation”, which includes several enzymes, including cytochrome oxidase subunit, NADH dehydrogenase subunit, and ATP synthase subunit. Proteins present in the “spliceosome” pathway were Hsp70, RNA-binding protein, and ATP-dependent RNA helicase DDX5/DBP2. Chitinase was the changed protein in “amino sugar and nucleotide sugar metabolism” pathway. Hsp70 and cyclic AMP-dependent transcription factor ATF-4 were the changed participants of the “protein processing in endoplasmic reticulum” pathway. Genes of some enzymes, such as acetyl-CoA synthetase, gluconolactonase, glucuronosyltransferase, alpha-glucosidase, DNA-directed RNA polymerase III subunit RPC1, glutathione S-transferase, glycerol-3-phosphate O-acyltransferase, formyltetrahydrofolate dehydrogenase, lysosomal acid phosphatase, porphobilinogen synthase, were also differentially expressed during the cold stress. KEGG Pathway Maps were provided in S1 File.

### QRT-PCR validation

**Fig 6:**
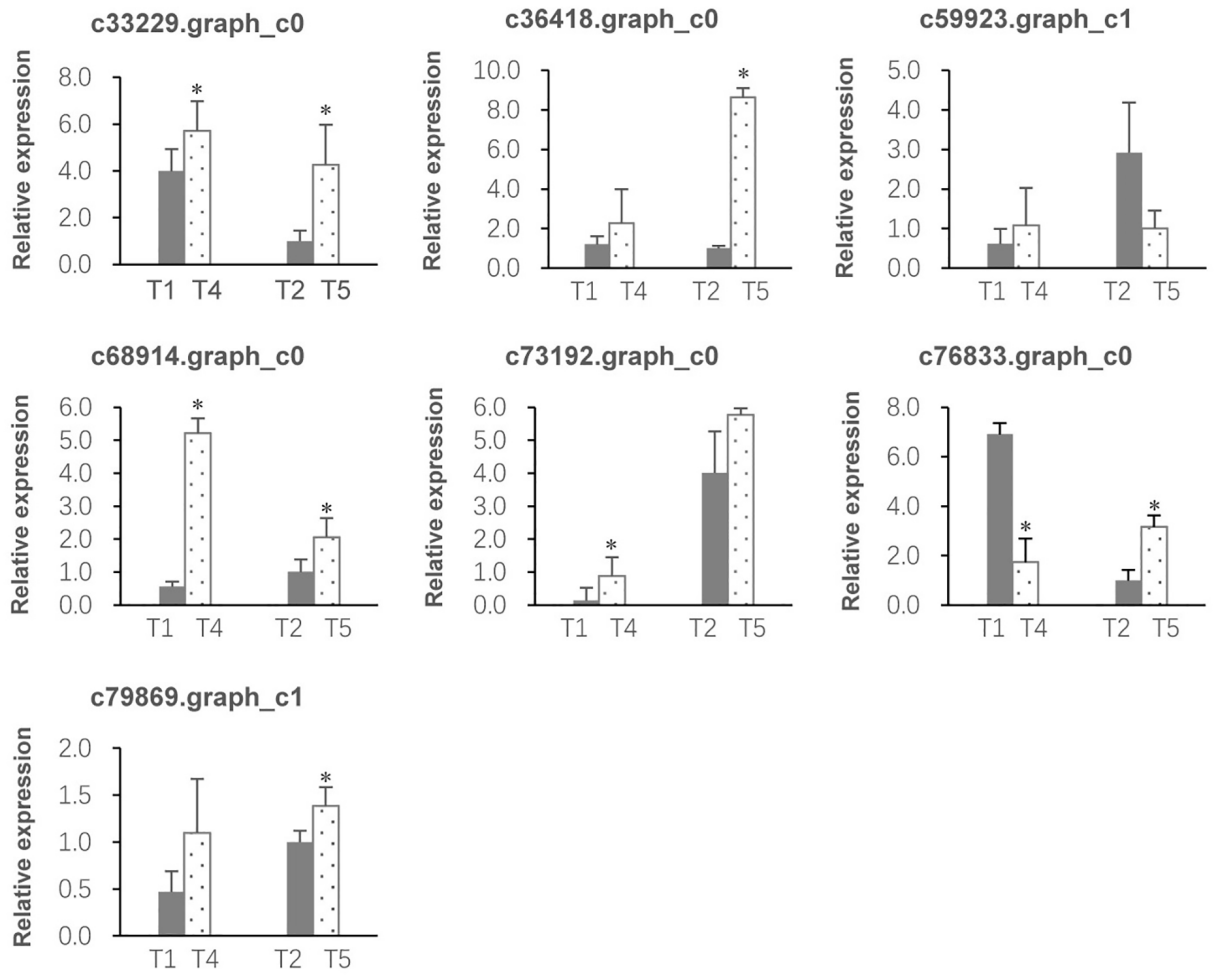
Validation of the DEGs by qRT-PCR. c33229.graph_c0~ c79869.graph_c1 are unigene IDs. T1&T2 represent the control groups; T4&T5 represent the low temperature groups. Relative expression values are presented as the mean ± SE of three biological replicates. Differences statistically significant are indicated by asterisk (P<0.05).

This experiment involved RT-PCR (reverse transcription PCR), gel electrophoresis, Sanger sequencing, and qPCR of seven common DEGs identified in the RNA sequence data. The non-redundant transcripts from the transcriptome data and the RT-PCR fragments showed high sequence identities, validating the accuracy of the Illumina sequencing and *de novo* assembly. The qPCR results are presented in Fig 6. It can be seen that expression changes were in the same direction for the qPCR, but the magnitude differed. The FKPM of these genes were shown in Table 3. In general, the results were consistent with the Illumina sequencing data, corroborating the reliability and accuracy of the transcriptome analysis. This ensures the feasibility and sustainability of our further research on these or other DEGs from the transcriptome data.

## Discussion

In this study, we conducted a comprehensive transcriptome analysis and characterized the gene expression profiles of *E*. *hippophaecolus* under cold stress. Through the analysis of DEGs sets, we identified transcriptome changes in *E*. *hippophaecolus* larvae.

From the assembly result, it can be seen that the length of 57.79% of the transcripts and 73.76% of the unigenes are less than 500 bp. N50 length of the transcripts is 1,687 bp, while the length of the unigenes is 904 bp. The completeness assessment by BUSCO indicates the comparatively high quality of the assembly according to the study of Simão et al. (2015) [41]. Our results are in line with other insect transcriptome projects using Illumina technology [36, 54].

Only 22.39% of the unigenes produced a significant blast hit to an arthropod sequence in the nr database. This can be partly attributed to the limited information available on Lepidoptera transcriptomes and genomes. Moreover, some genes in this moth could be highly diverged, thereby excessing the blast threshold. Unsurprisingly, most of the *E*. *hippophaecolus* unigenes matched sequences from other lepidopteran species.

In this study, DEG libraries from cold treated and untreated *E*. *hippophaecolus* larvae were compared to identify functional genes at the whole-organism level. There were small-scale changes in gene expression. The number of DEGs from the T1-T4 comparison was larger than in the T2-T5 comparison. This difference may due to the different stages of larvae. Young larvae of *E*. *hippophaecolus* may not be as innately cold tolerant as older larvae to resist cold, so they may have to regulate related genes intensively to compensate. It has been previously noted in *Drosophila montana* that genes implicated in increasing cold tolerance are different between diapausing and non-diapausing flies, and that this could be due to different energy budgets at different life stages [55]. There were seven DE genes in common between developmental stages. Little previous work has been conducted on these genes, but our work suggests they are important for cold tolerance. Although there was little overlap in the genes between stages, there were some overlaps in the processes involved. Processes such as “binding”, “RNA binding”, “structural constituent of cuticle”, “N-acetylmuramoyl-L-alanine amidase activity” and “peptidoglycan catabolic process”, in which genes involved were all up-regulated; “nucleic acid binding” and “nucleic acid binding transferase activity” in which genes involved were all down-regulated were found in common through GO analysis.

From the pathway analysis, we found some *hsps* were differentially expressed. HSPs are well-known chaperone proteins that play important roles in cold hardiness and organism responses to other stressors (e.g., heat, low oxygen levels, UV radiation, bacterial and viral infection, heavy metals) that can affect the folding and functional conformation of proteins [56, 57]. That not all HSP genes were upregulated is consistent with previous studies [58, 59] and highlights that the mechanisms regulating these genes need further research. The “oxidative phosphorylation” pathway, which involves several enzymes, including cytochrome oxidase subunit, NADH dehydrogenase subunit, and ATP synthase subunit, was significantly downregulated under cold treatment. Antioxidant defense is a cell preservation strategy in response to environmental challenges [60] (e.g., heat, cold, UV radiation, osmotic challenge, toxins). Genes associated with the “metabolism of xenobiotics by cytochrome P450” pathways were modestly downregulated. It has been previously reported that cytochrome P-450 is related to temperature regulation [61].

In addition, genes for a number of cuticular proteins were also upregulated. These proteins are essential during insect cuticle formation and in the development of insect body structures and organs. Previous studies have shown that some insects upregulate the expression of cuticular protein genes to resist cold or other environmental stressors [19, 62–64]. To investigate the changes of structure or amount of the cuticle in response to cold could be an avenue for future work.

In this research, we also discovered many DEGs and the roles they play in cold tolerance in insects are unknown. Though it is hard to elucidate the molecular mechanisms underlying a complex process from the action of a few genes, the functions of these genes are worth being verified in future studies.

Curiously, the proteins most associated with insect cold hardiness, AFPs, were not found in the transcriptome profile. There may be several reasons for this. Firstly, although AFPs have been well studied, only about 200 insect AFP genes have been registered in GenBank. Secondly, the relationship between mRNA transcript levels and protein levels is complex. Integrated proteome/transcriptome studies have shown that mRNA levels are poorly associated with protein levels for all but the most abundant proteins [65]. Thirdly, it may be that more genes/proteins would be stimulated or expressed in the chill recovery phase [56]. Fourthly, genes involved in increasing cold tolerance have been shown to differ even between closely related species [66]. Finally, it is generally presumed that AFPs play a greater role in the cold resistance of freeze intolerant insects; for freeze tolerant species, on the other hand, AFPs may not be important [12].

During the transcriptome construction, we took into account the development stage of the moth, but neglected the replicates, thus might cause problems such as false positives when conducted DE analysis. Mixing samples in one RNA-seq sample may cause inconsistent of gene expression. The parallel experiment within different development stages could provide a reference when we choose interesting genes, thus we focus on the common DEGs in the parallel tests. Moreover, further validation results of the DEGs using qRT-PCR showed the consistency of the transcriptome data. This study provides a profile of the candidate genes that are suspected to be involved with insect cold hardness. Many further studies should be carried out to test and verify the function the candidate genes.

In conclusion, this study is the first to characterize the transcriptome of *E*. *hippophaecolus* larvae without a reference genome. Our data will facilitate future genomic research and in-depth annotation of insect genomes. Moreover, the preliminary identification of genes and pathways exhibiting differential expression under cold stress may further our general understanding of cold resistance in insects.

## Supporting information

S1 Fig Statistics of *de novo* assembly of the transcriptome. (a) Distribution of all unigenes length. (b) Distribution of all transcripts lengths. The x-axis indicates the length and y-axis indicates the number of unigenes or transcripts.
(TIF)

S2 Fig Characteristics of the homology search of Illumina sequences in the nr database. (TIF)

S3 Fig Correlation between samples. (TIF)

S4 Fig Correlations matrix heat map. (TIF)

S1 Table The list of the sequences identified as ncRNA. (XLSX)

S2 Table Primers for real-time PCR. (PDF)

S3 Table Part of the sequencing and assembly results. (DOCX)

S4 Table The blast information for each contig. (XLSX)

S5 Table DE information for each contig. FDR: false discovery rate; Log_2_FC: log_2_ fold change of DE genes; regulated: the expression level of DEGs in T04/T05 compared to T01/T02, including three situations, up-regulated, down-regulated or normal; T01~T05 are FPKM values of unigenes in each sample.
(XLSX)

S6 Table Annotations of the DEGs. _FPKM: FPKM values of unigenes in each sample; FDR: false discovery rate; Log_2_FC: log_2_ fold change of DE genes; regulated: the expression level of DEGs in T04/T05 compared to T01/T02, including three situations, up-regulated, down-regulated or normal; other columns are functional annotations of unigenes.
(XLSX)

S1 File KEGG pathway maps of DEGs in T1-T4. (ZIP)
