# Supplementary figures and images for: Differential transcriptome analysis reveals genes related to cold tolerance in seabuckthorn carpenter moth, Eogystia hippophaecolus
Source: PLoS One. 2017 Nov 13;12(11):e0187105. doi: 10.1371/journal.pone.0187105 (PMC5683614; doi:10.1371/journal.pone.0187105)

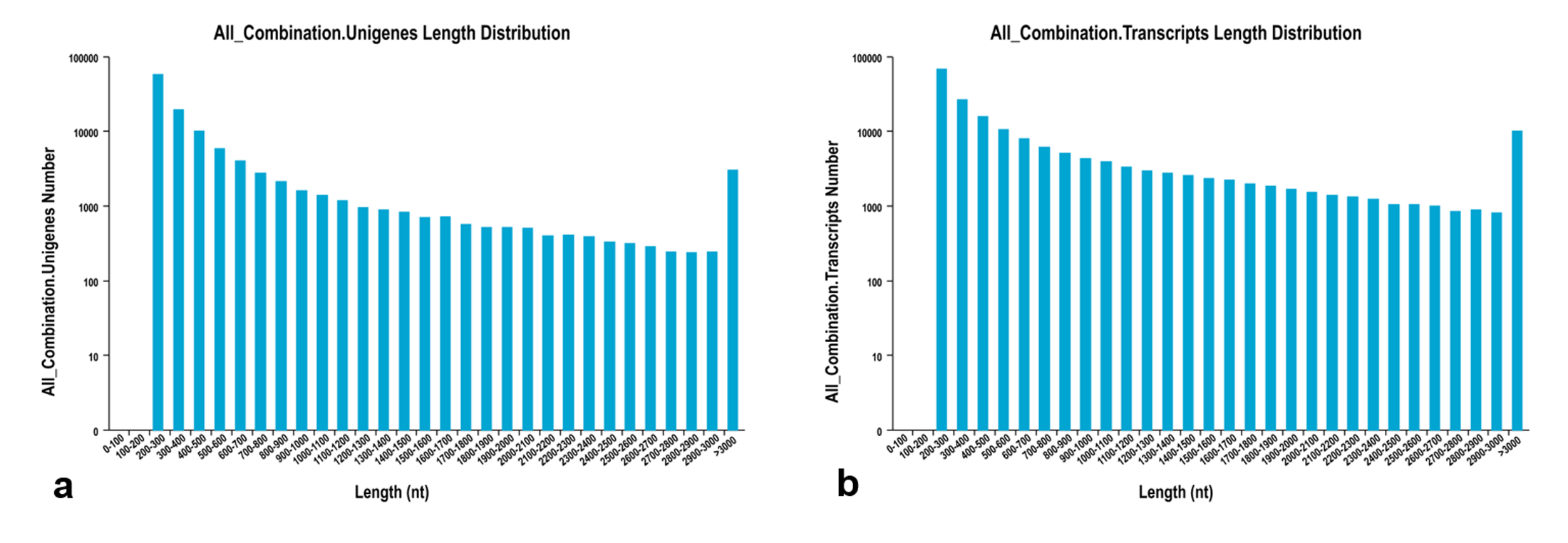

Supplement: S1 Fig — (a) Distribution of all unigenes length. (b) Distribution of all transcripts lengths. The x-axis indicates the length and y-axis indicates the number of unigenes or transcripts. (TIF) [file pone.0187105.s001.tif]

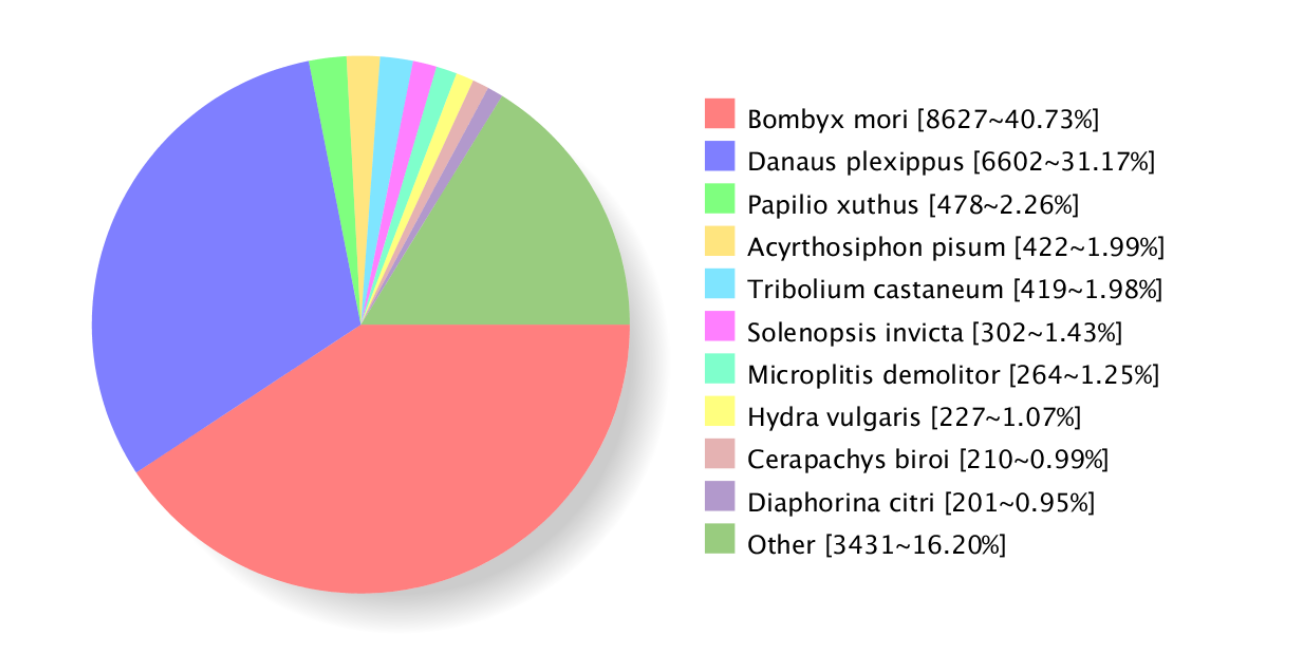

Supplement: S2 Fig — (TIF) [file pone.0187105.s002.tif]

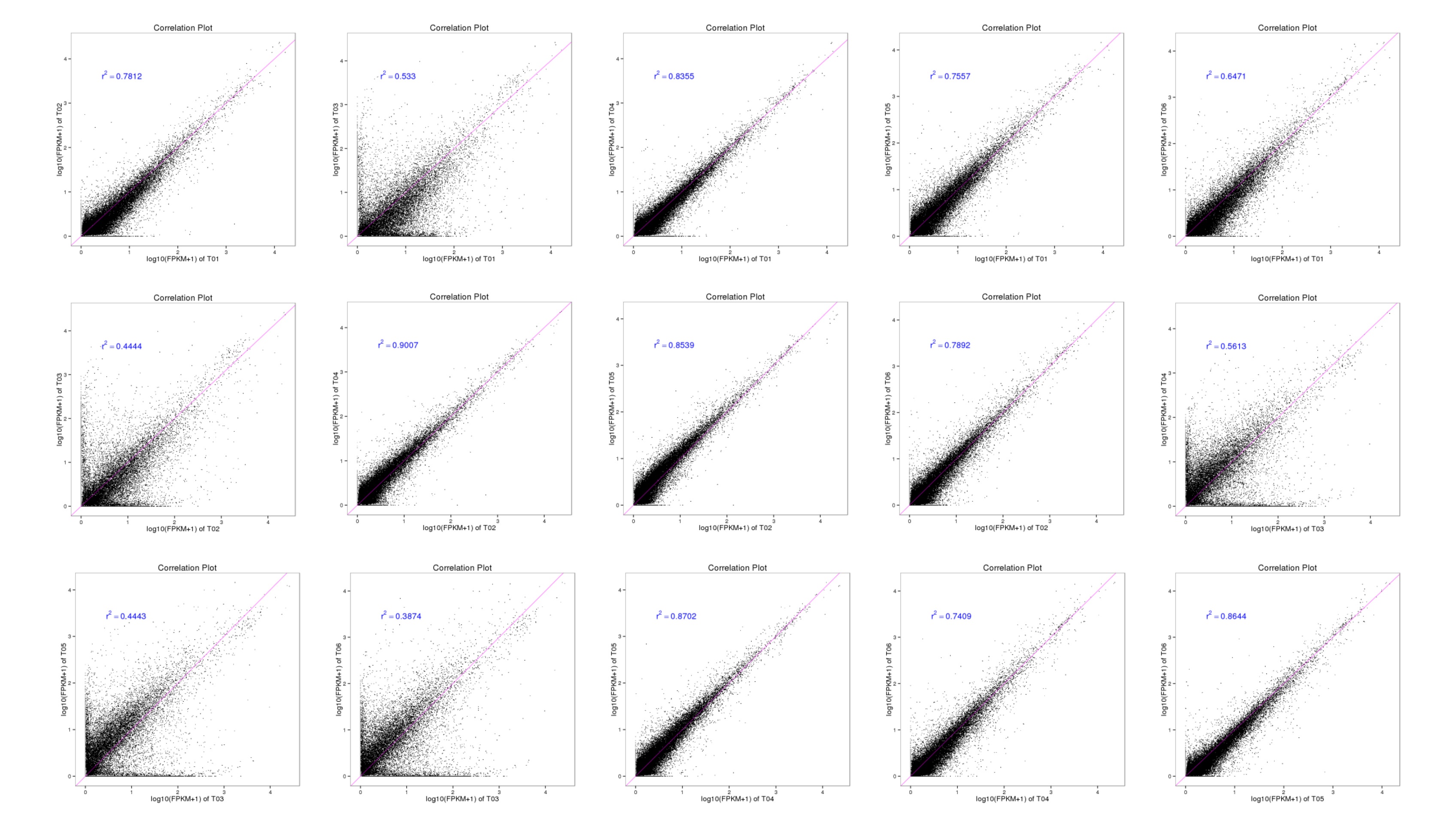

Supplement: S3 Fig — (TIF) [file pone.0187105.s003.tif]

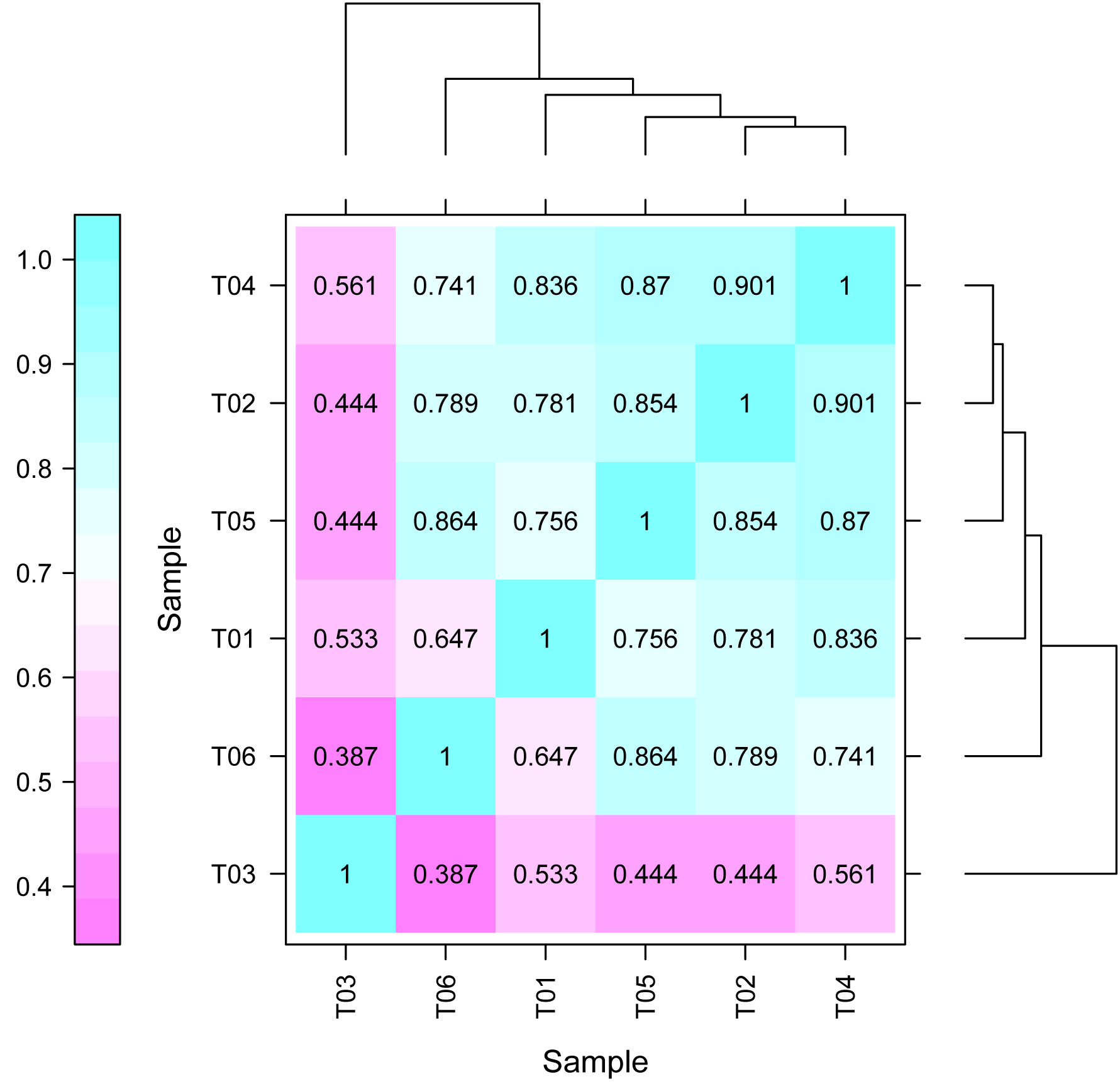

Supplement: S4 Fig — (TIF) [file pone.0187105.s004.tif]

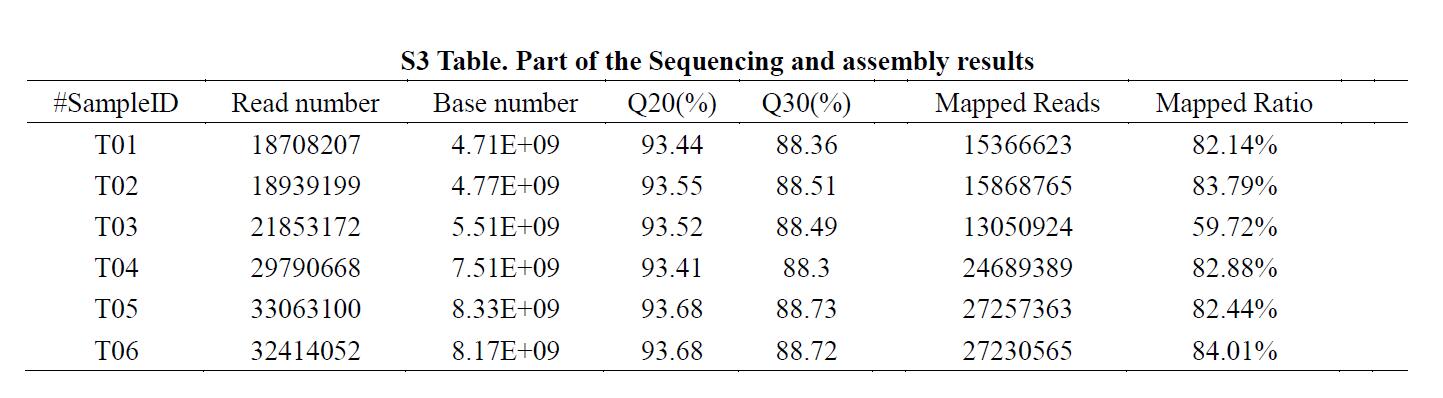

Supplement: S3 Table — (DOCX) [file pone.0187105.s007.docx]

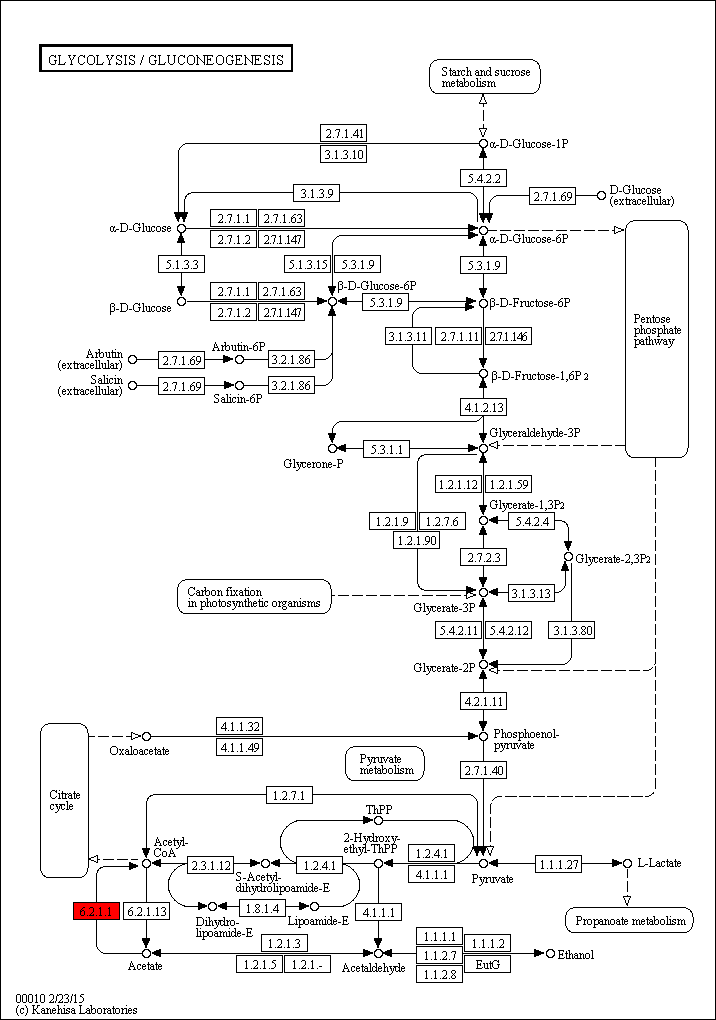

Supplement: S1 File — (ZIP) [file pone.0187105.s011.zip › kegg_map/ko00010.png]

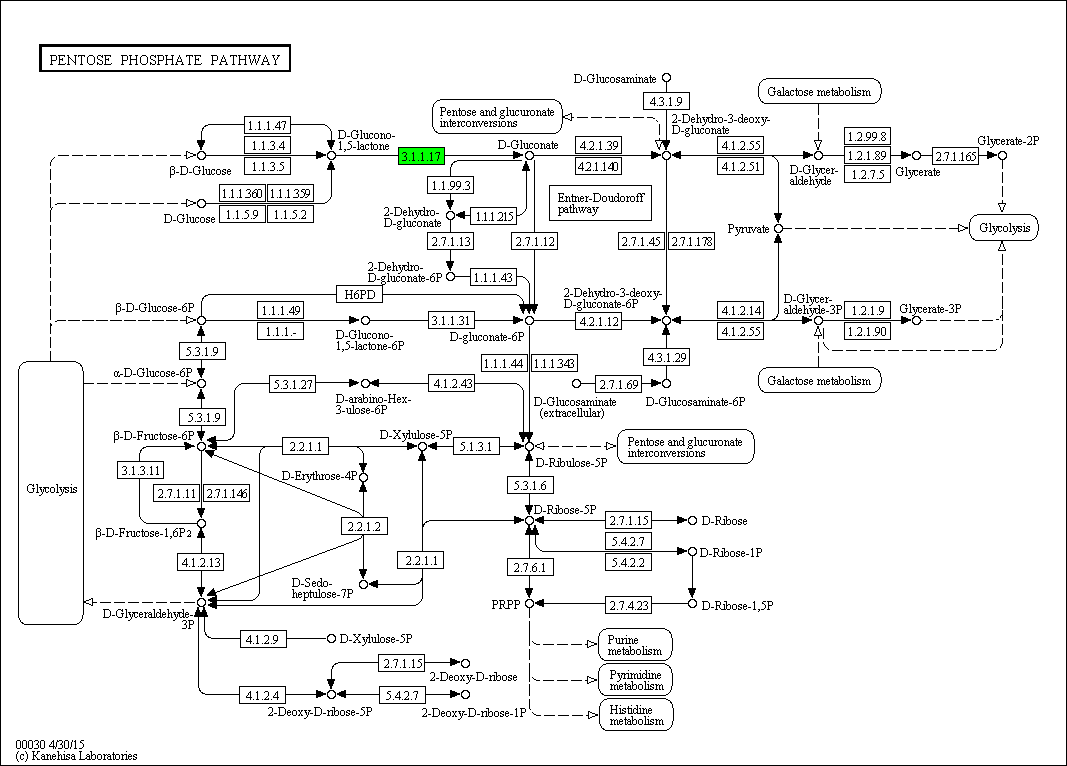

Supplement: S1 File — (ZIP) [file pone.0187105.s011.zip › kegg_map/ko00030.png]

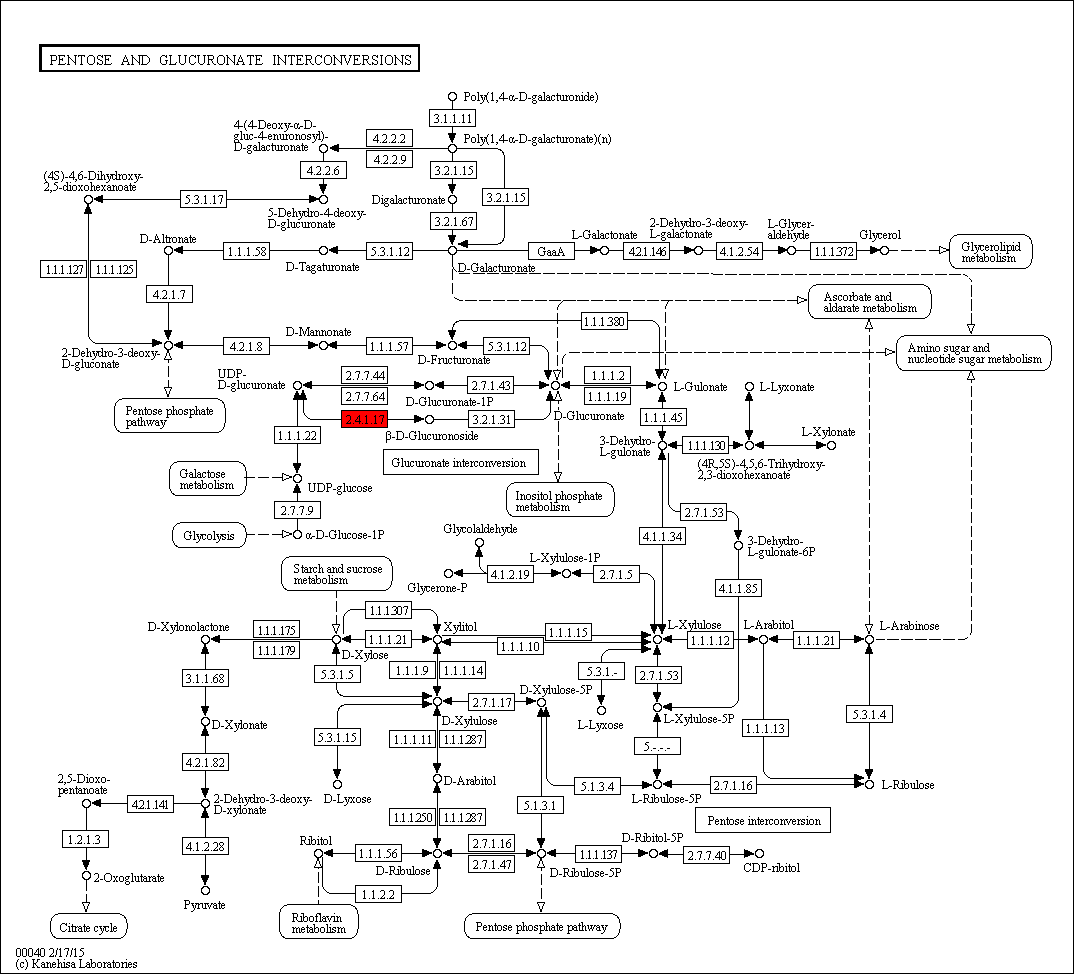

Supplement: S1 File — (ZIP) [file pone.0187105.s011.zip › kegg_map/ko00040.png]

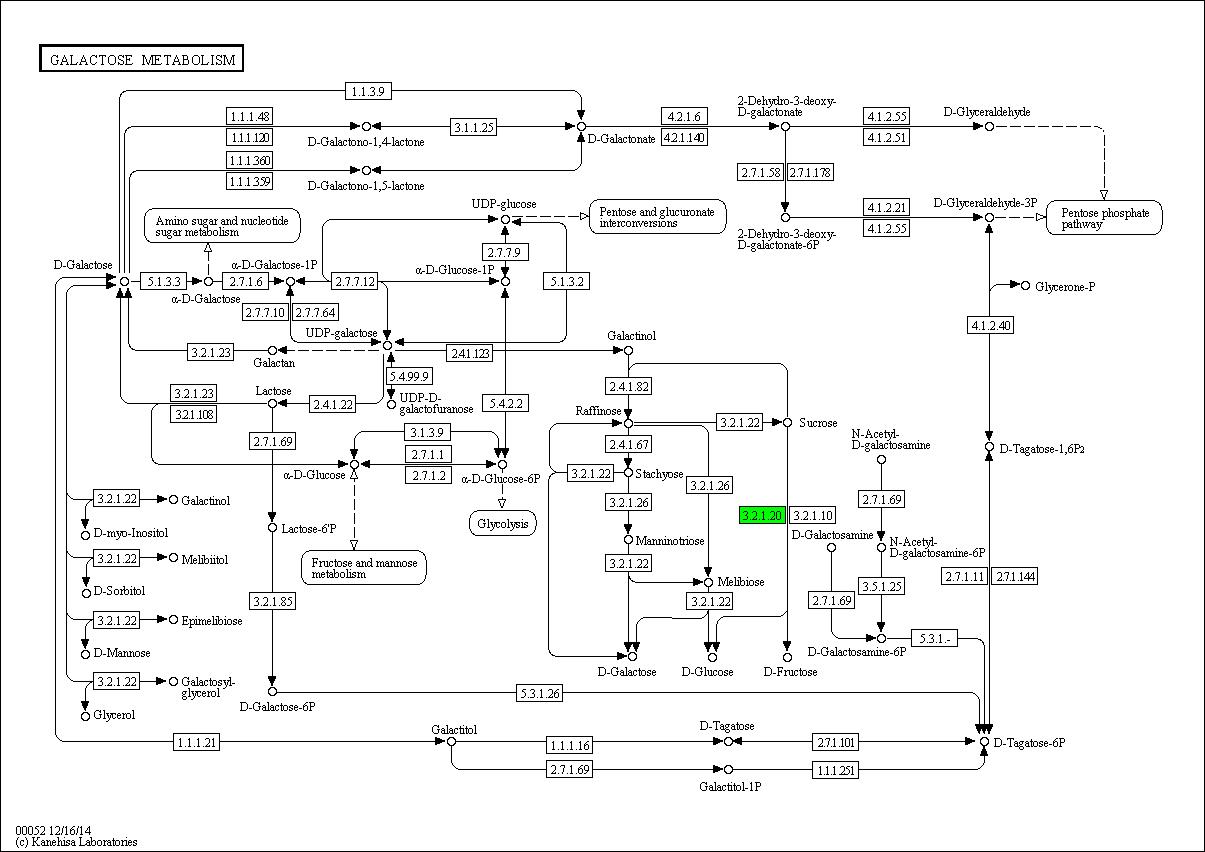

Supplement: S1 File — (ZIP) [file pone.0187105.s011.zip › kegg_map/ko00052.png]

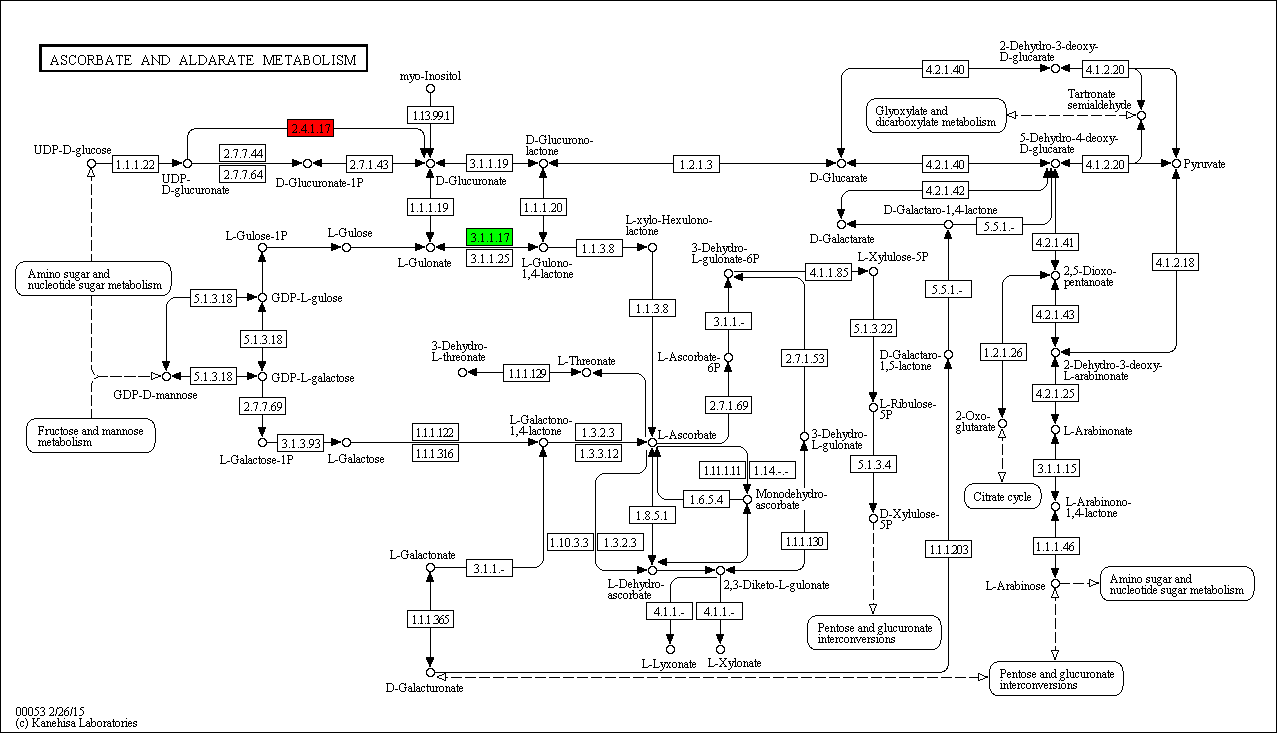

Supplement: S1 File — (ZIP) [file pone.0187105.s011.zip › kegg_map/ko00053.png]

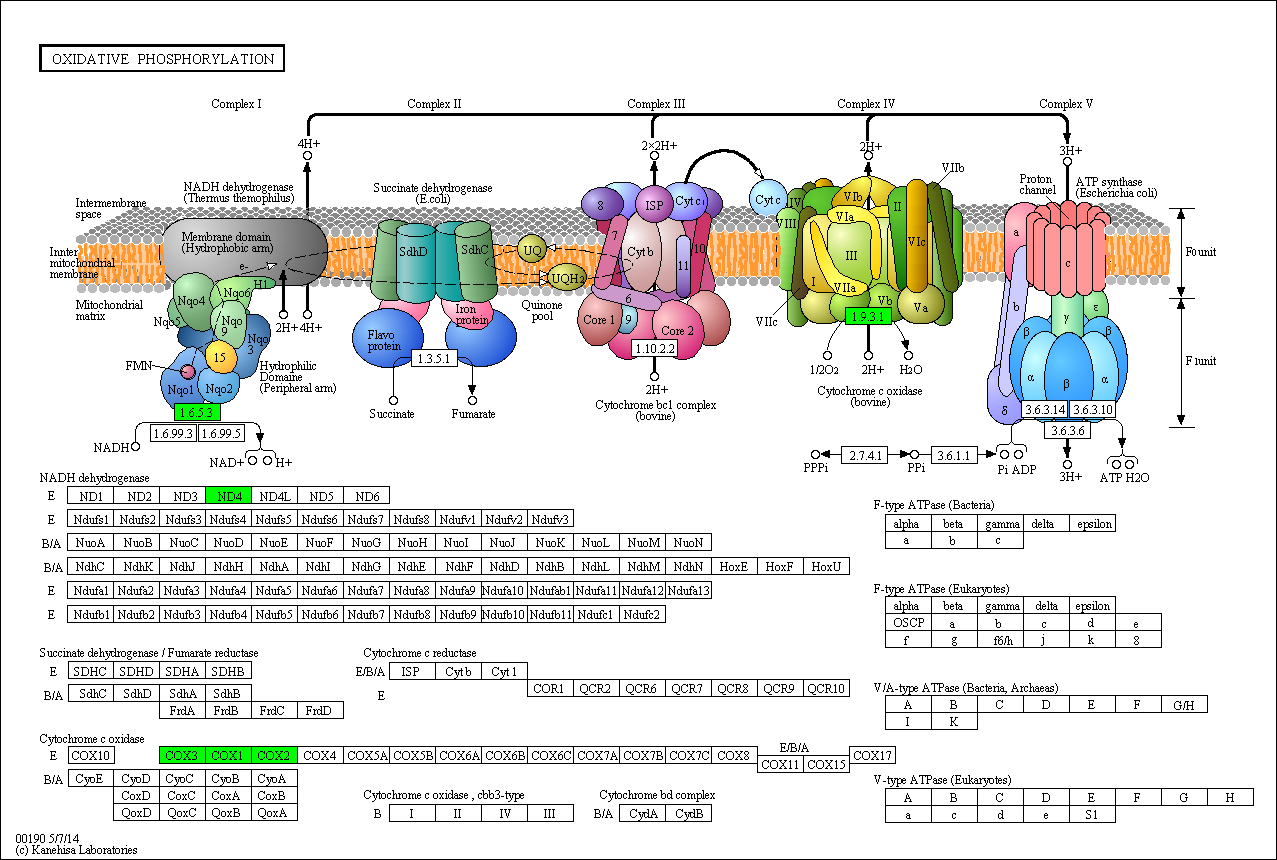

Supplement: S1 File — (ZIP) [file pone.0187105.s011.zip › kegg_map/ko00190.png]

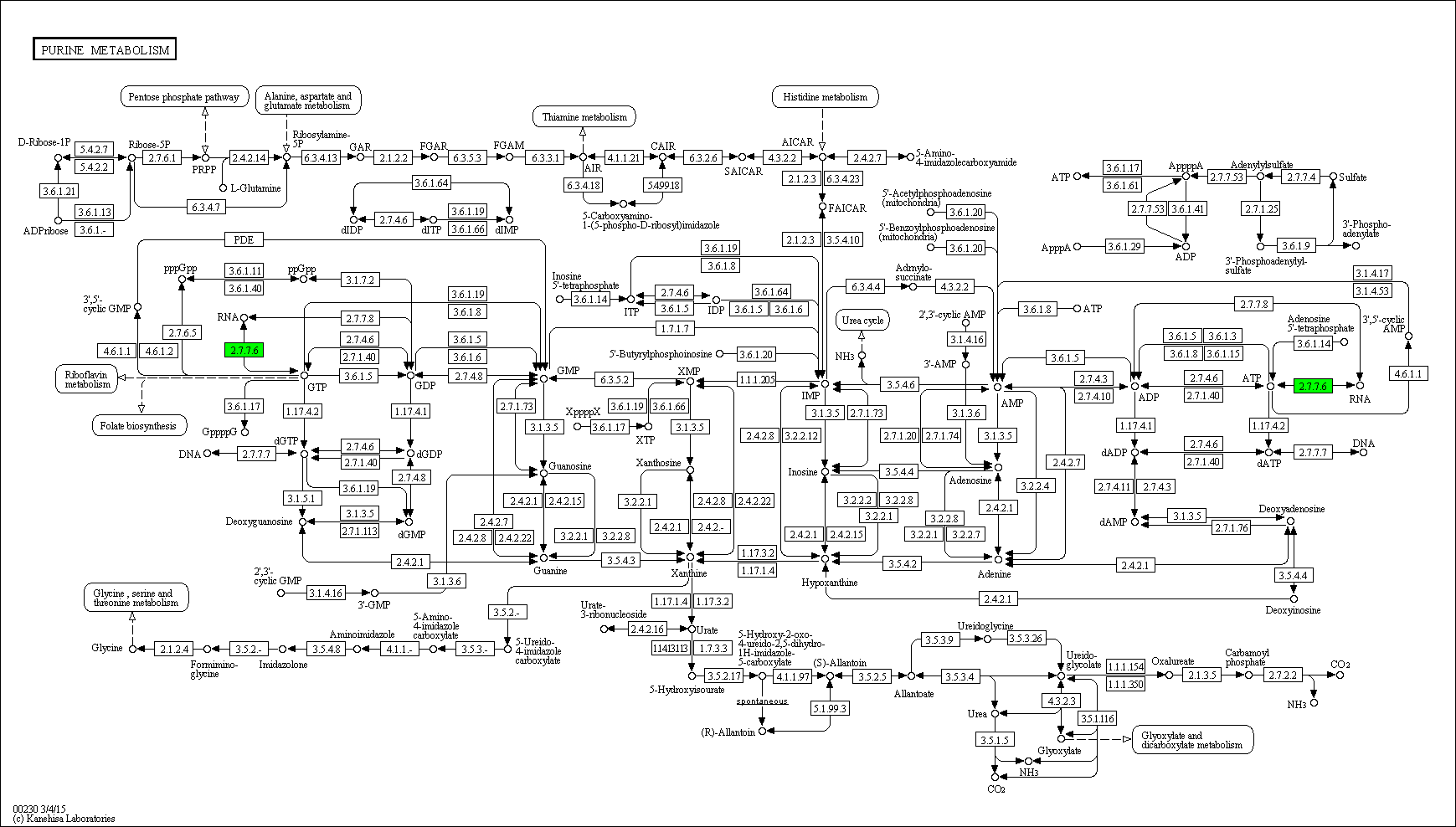

Supplement: S1 File — (ZIP) [file pone.0187105.s011.zip › kegg_map/ko00230.png]

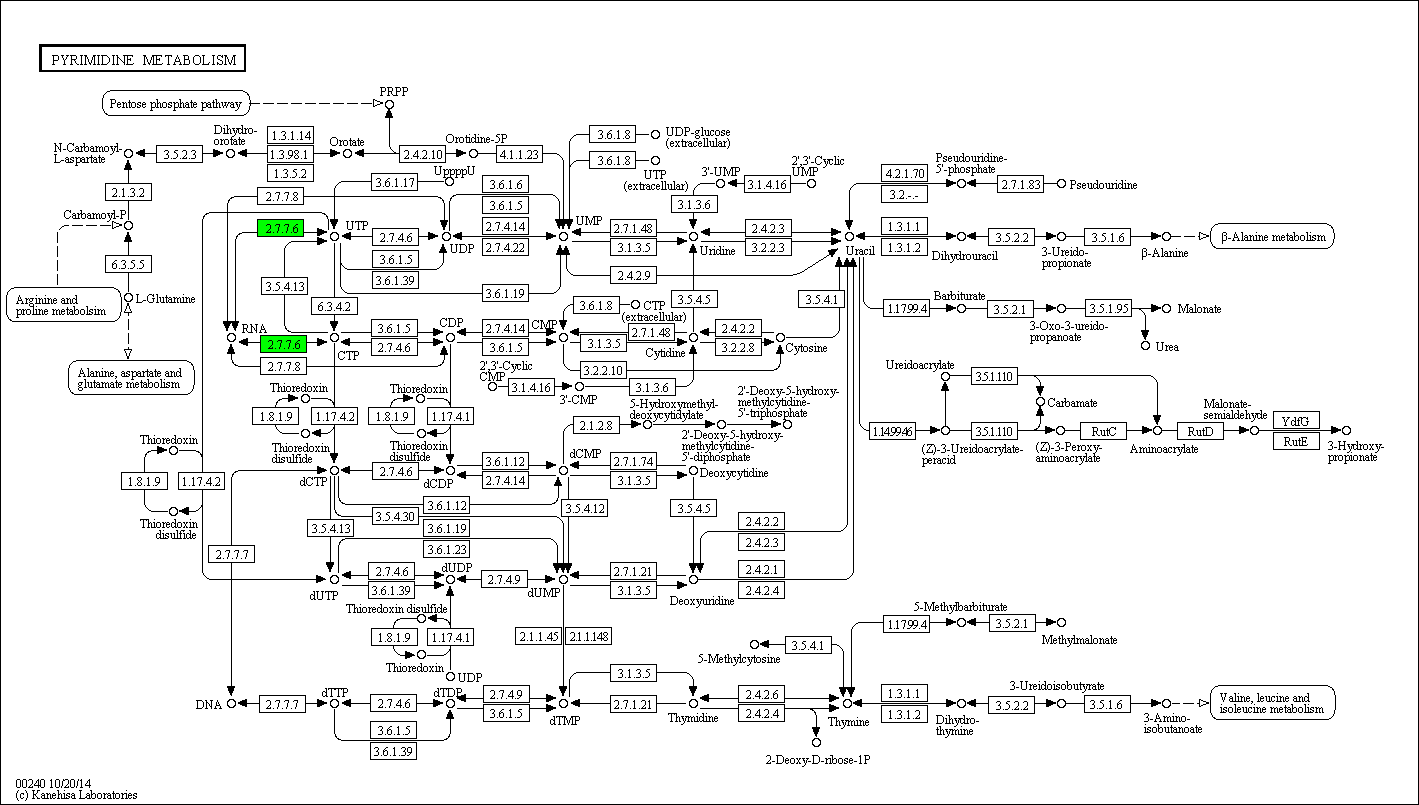

Supplement: S1 File — (ZIP) [file pone.0187105.s011.zip › kegg_map/ko00240.png]

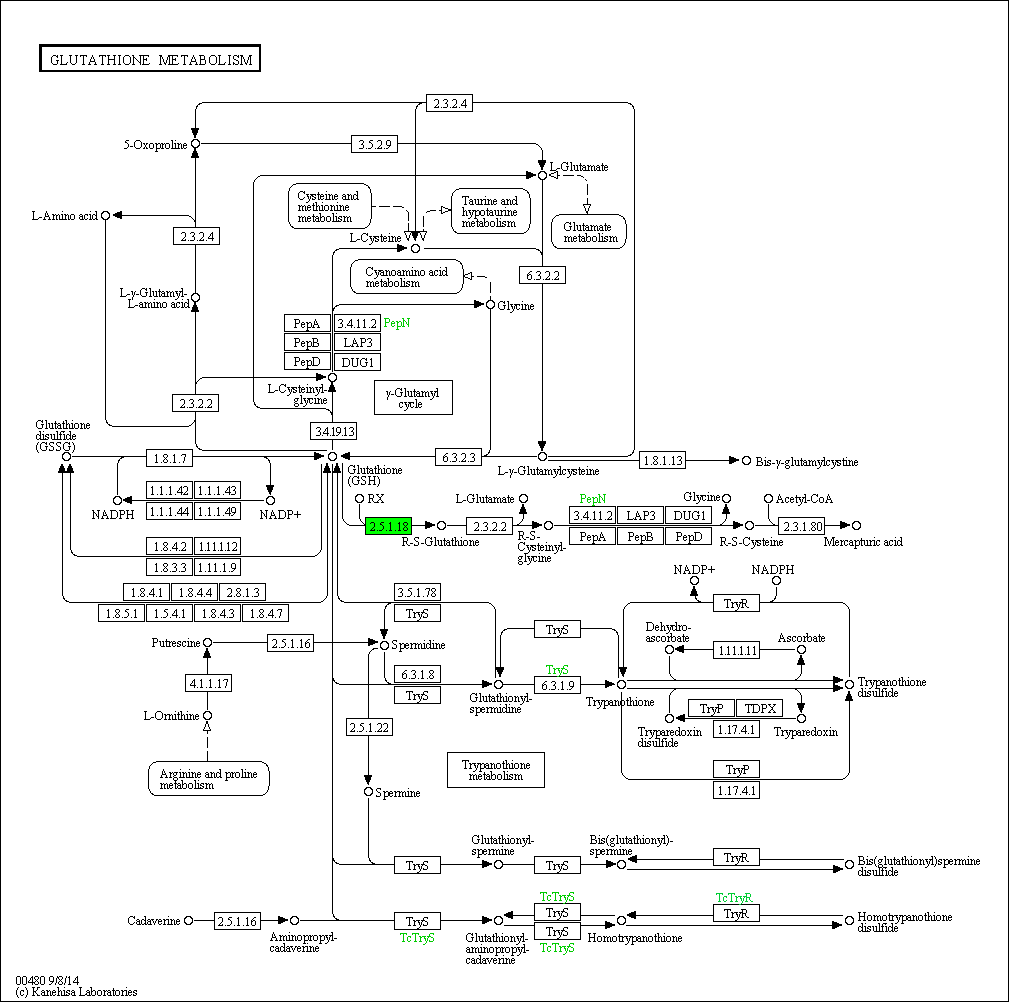

Supplement: S1 File — (ZIP) [file pone.0187105.s011.zip › kegg_map/ko00480.png]

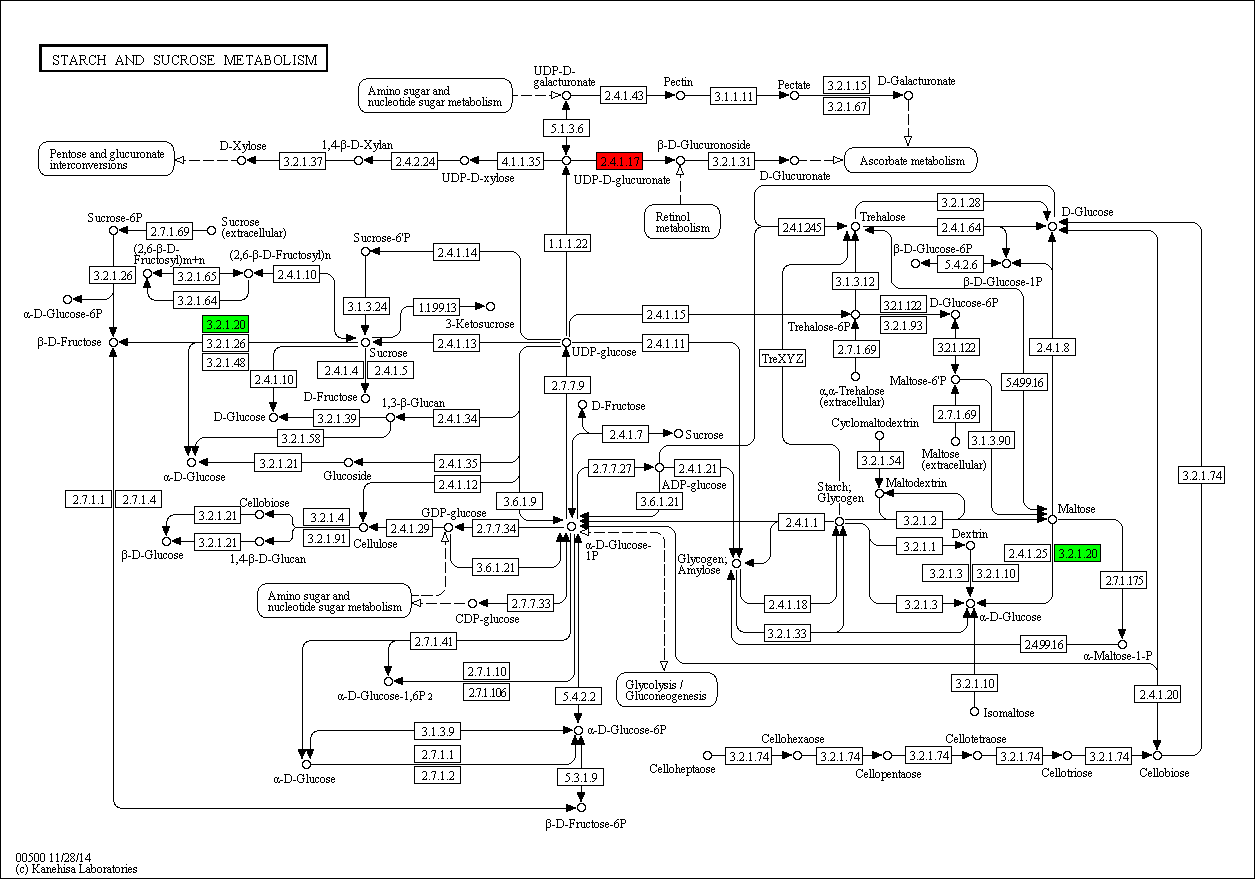

Supplement: S1 File — (ZIP) [file pone.0187105.s011.zip › kegg_map/ko00500.png]

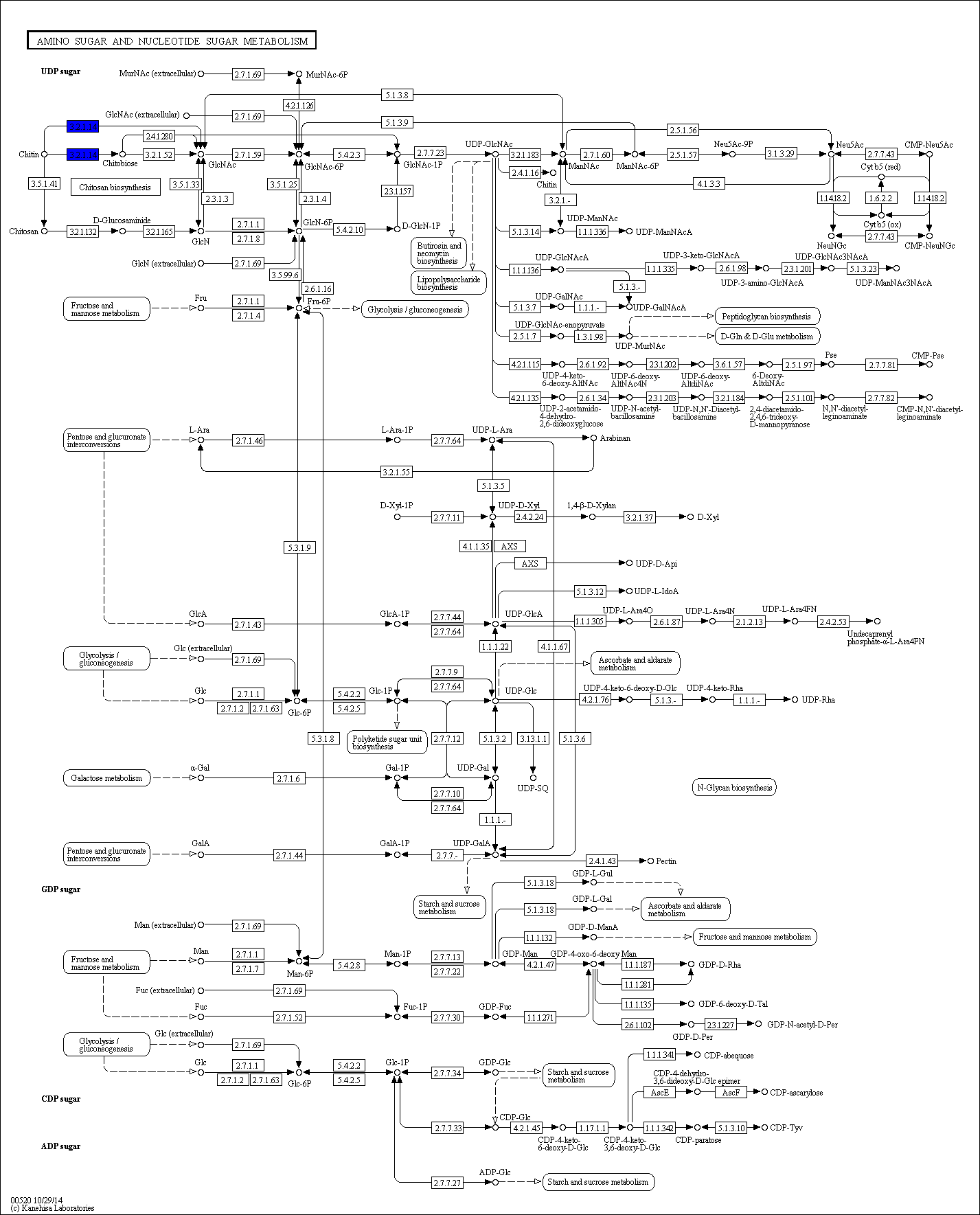

Supplement: S1 File — (ZIP) [file pone.0187105.s011.zip › kegg_map/ko00520.png]

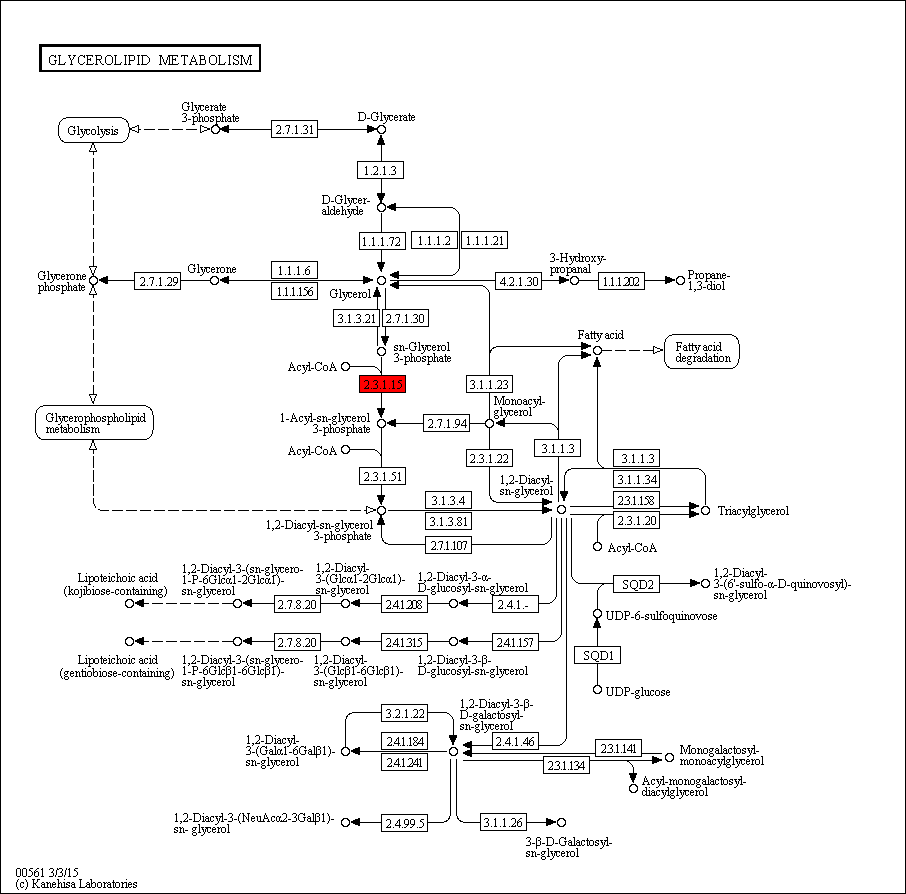

Supplement: S1 File — (ZIP) [file pone.0187105.s011.zip › kegg_map/ko00561.png]

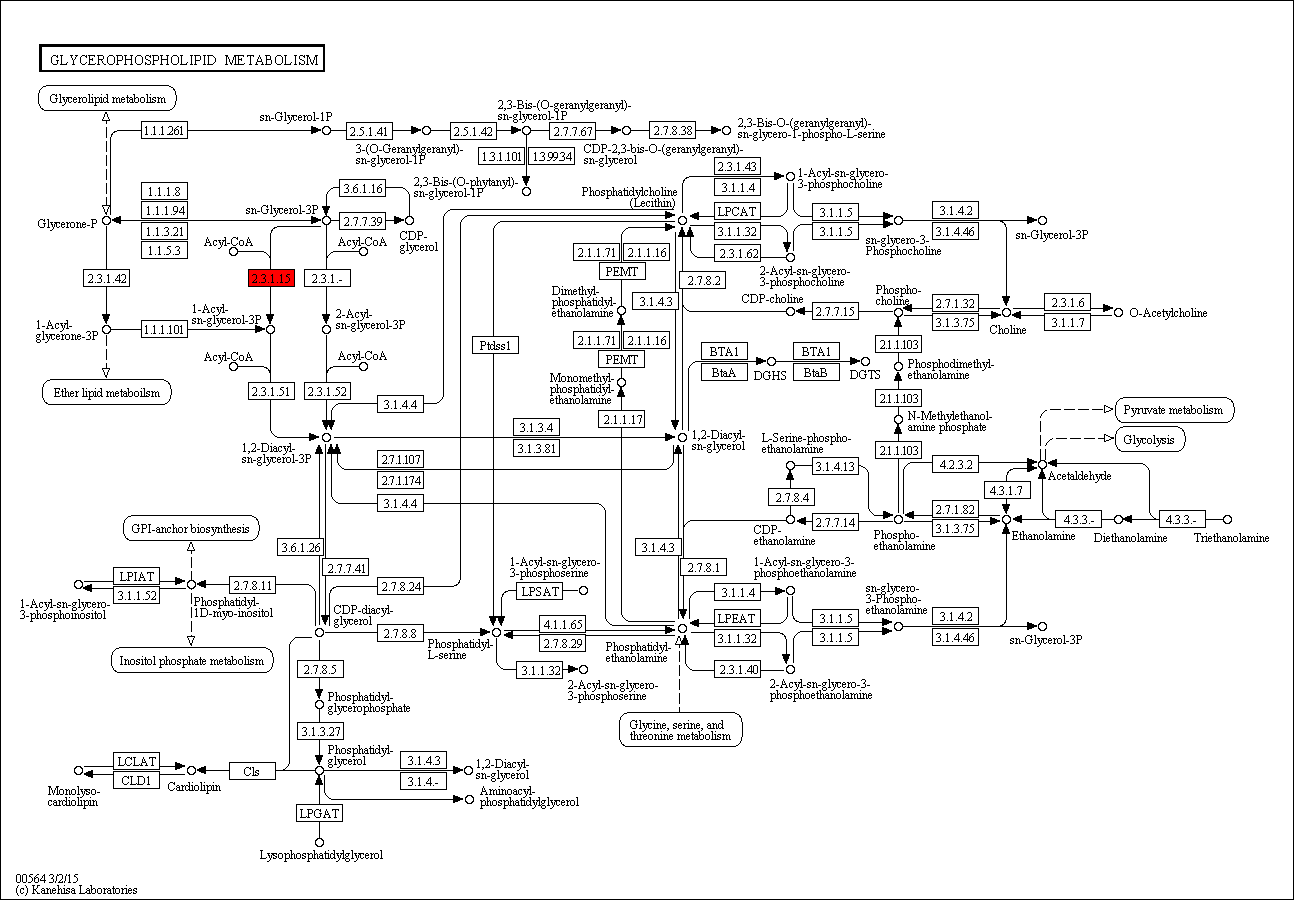

Supplement: S1 File — (ZIP) [file pone.0187105.s011.zip › kegg_map/ko00564.png]

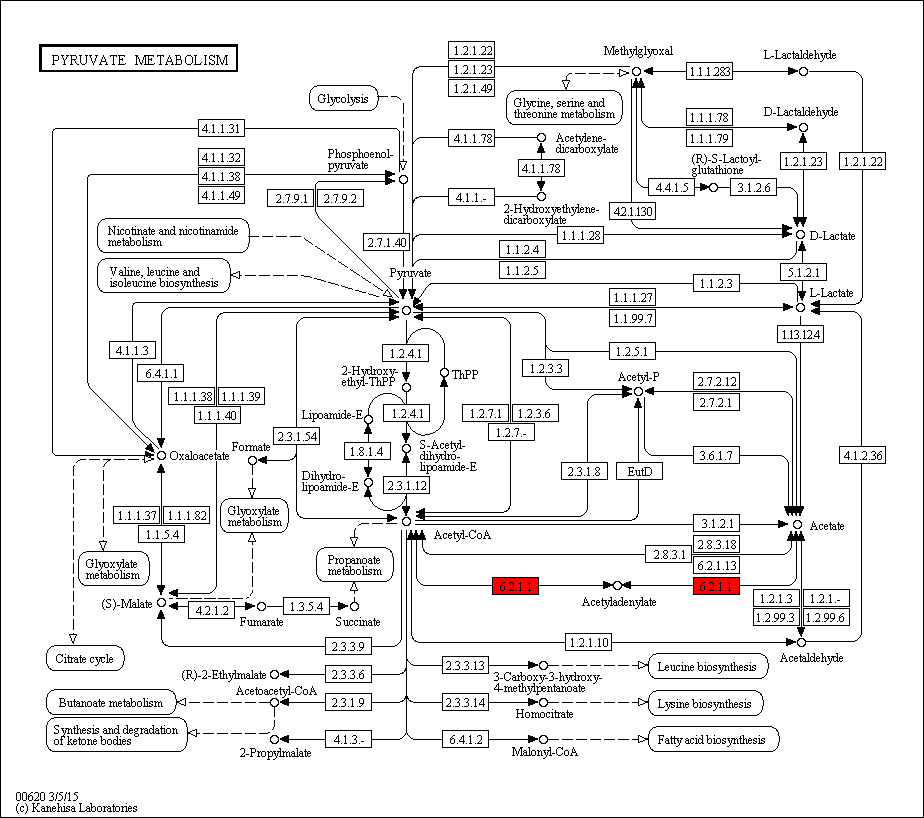

Supplement: S1 File — (ZIP) [file pone.0187105.s011.zip › kegg_map/ko00620.png]

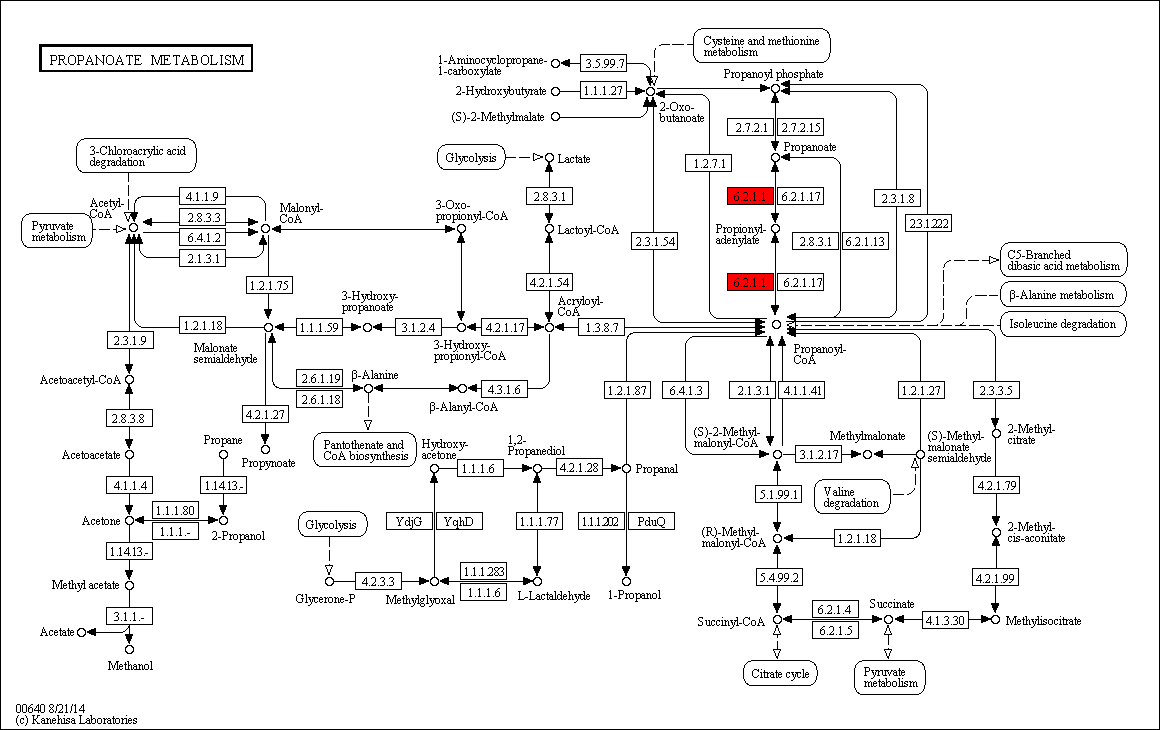

Supplement: S1 File — (ZIP) [file pone.0187105.s011.zip › kegg_map/ko00640.png]

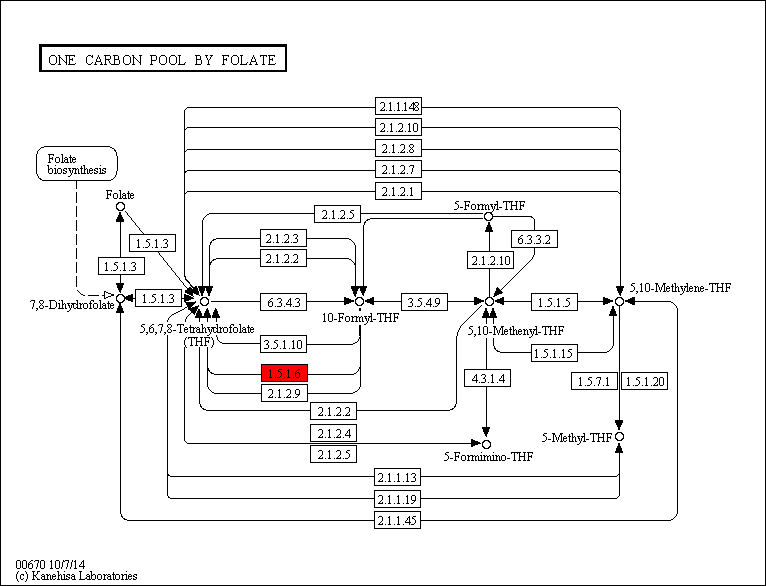

Supplement: S1 File — (ZIP) [file pone.0187105.s011.zip › kegg_map/ko00670.png]

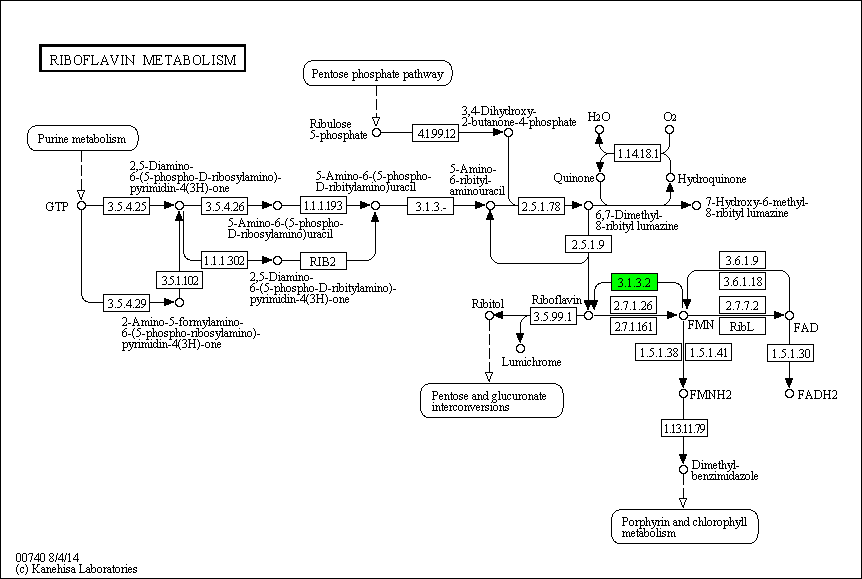

Supplement: S1 File — (ZIP) [file pone.0187105.s011.zip › kegg_map/ko00740.png]

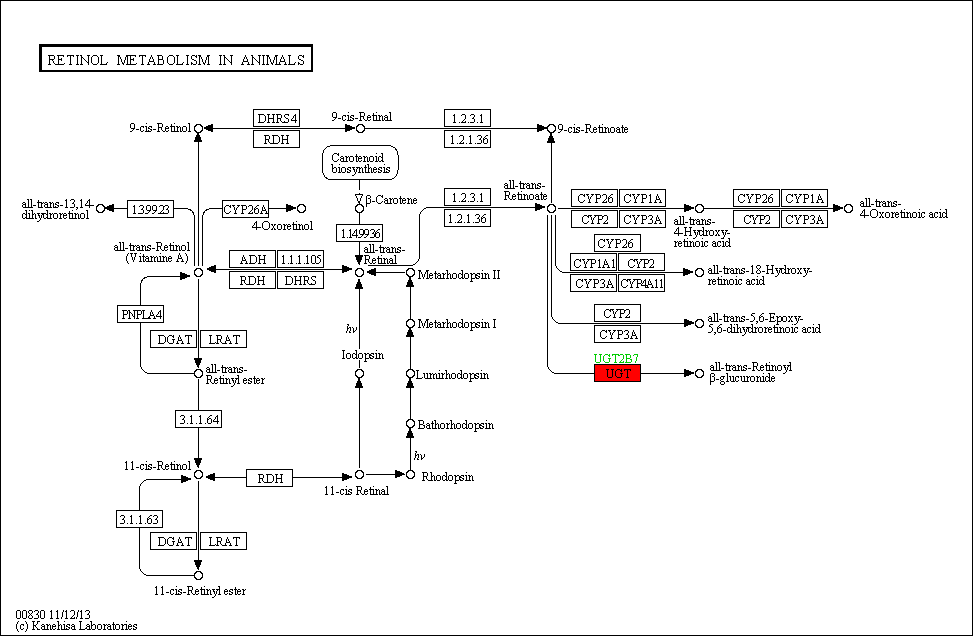

Supplement: S1 File — (ZIP) [file pone.0187105.s011.zip › kegg_map/ko00830.png]

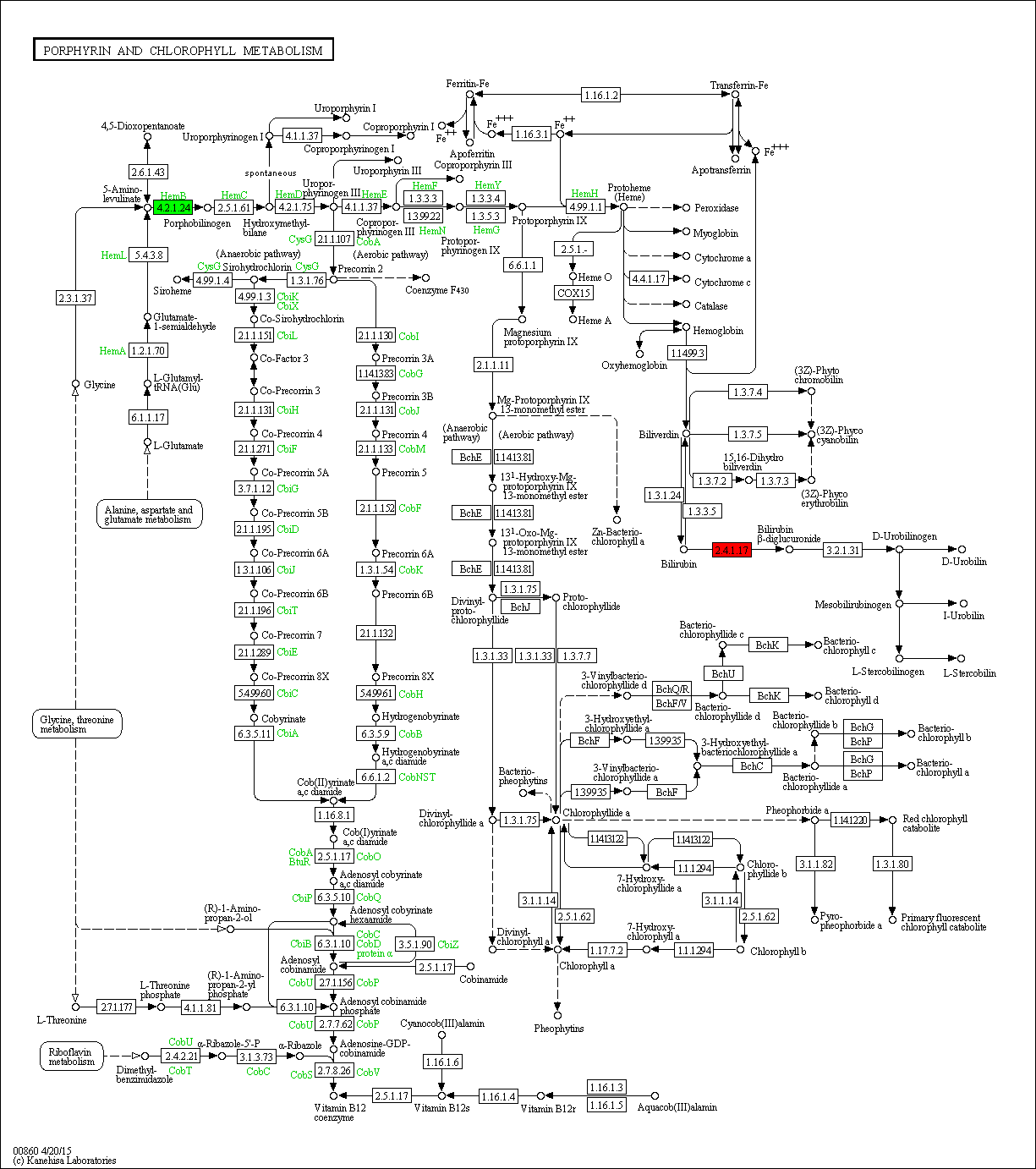

Supplement: S1 File — (ZIP) [file pone.0187105.s011.zip › kegg_map/ko00860.png]

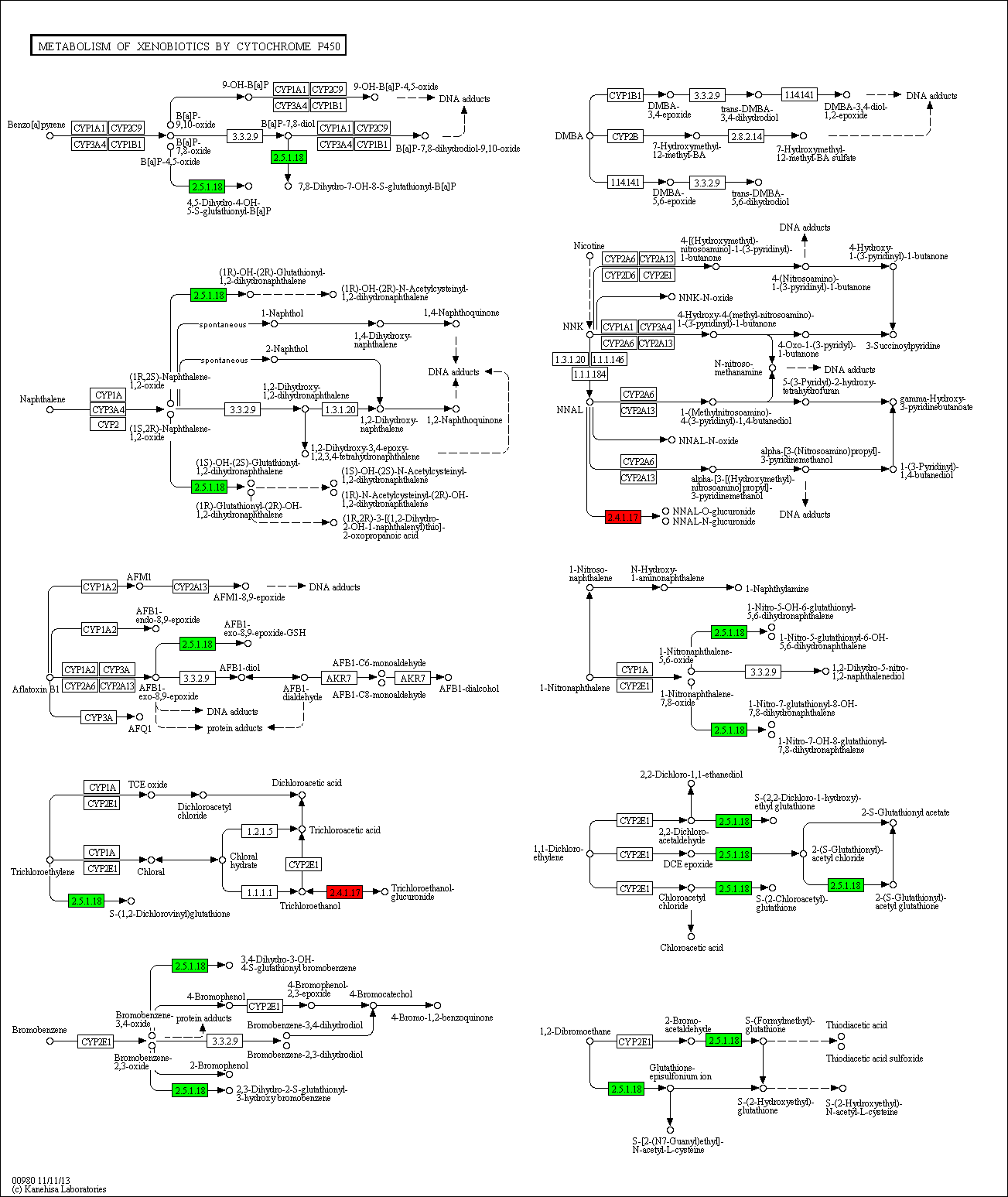

Supplement: S1 File — (ZIP) [file pone.0187105.s011.zip › kegg_map/ko00980.png]

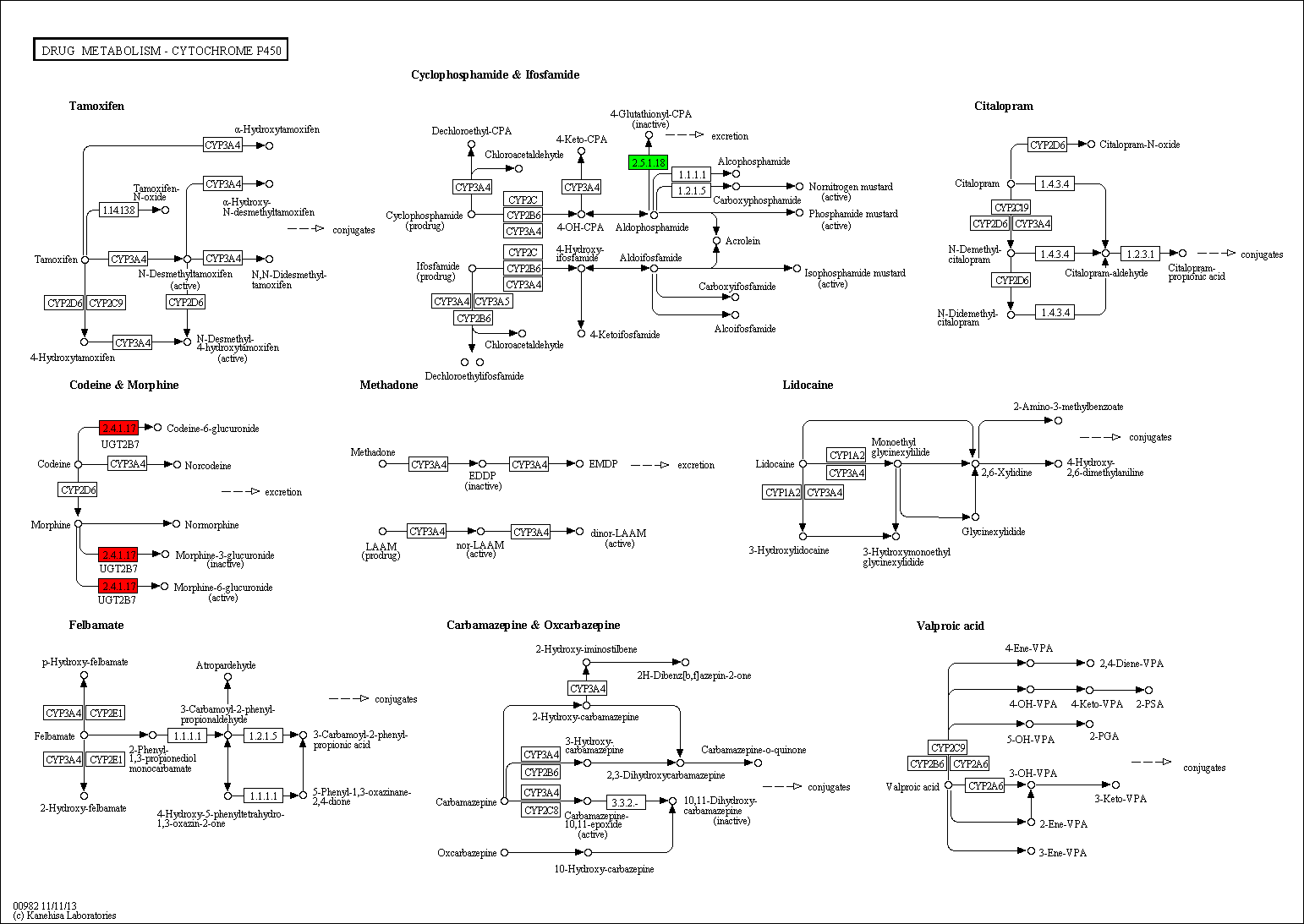

Supplement: S1 File — (ZIP) [file pone.0187105.s011.zip › kegg_map/ko00982.png]

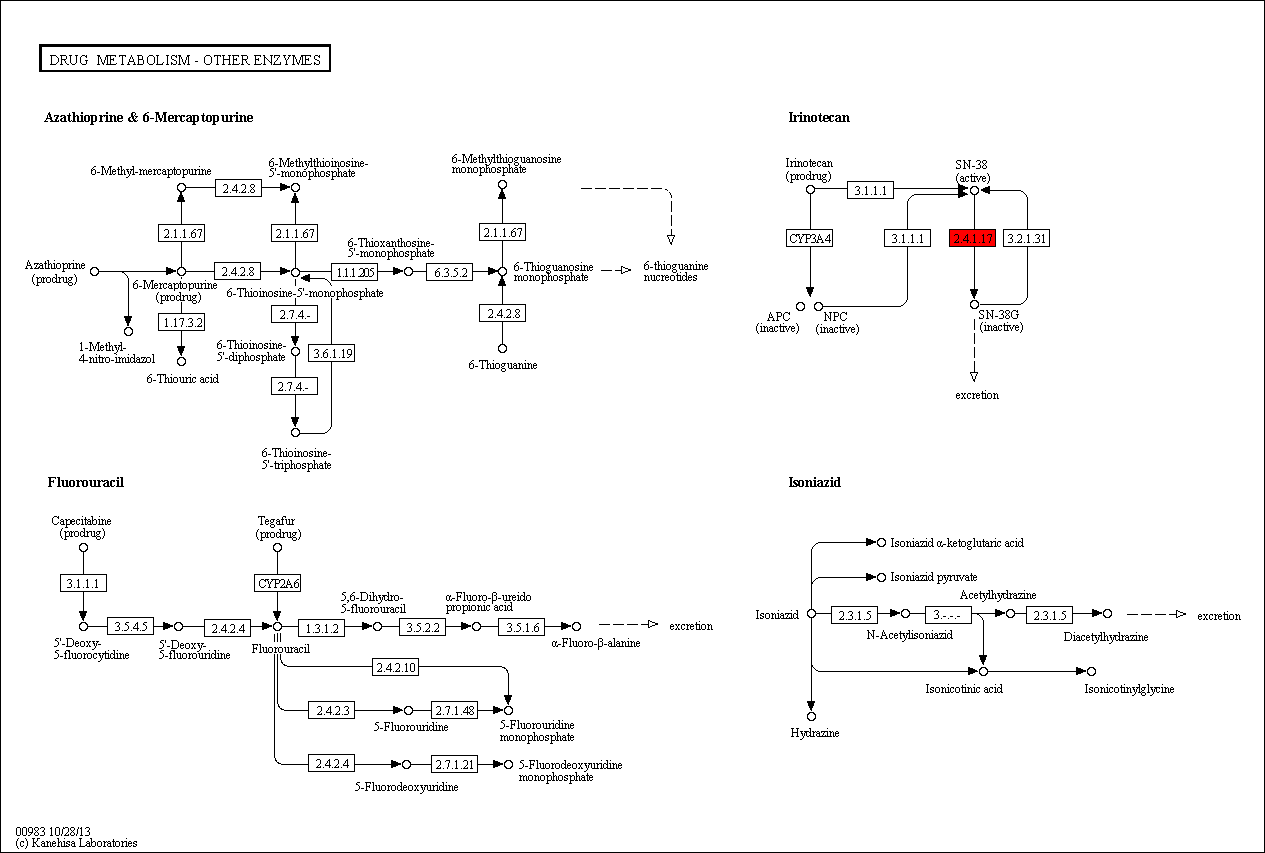

Supplement: S1 File — (ZIP) [file pone.0187105.s011.zip › kegg_map/ko00983.png]

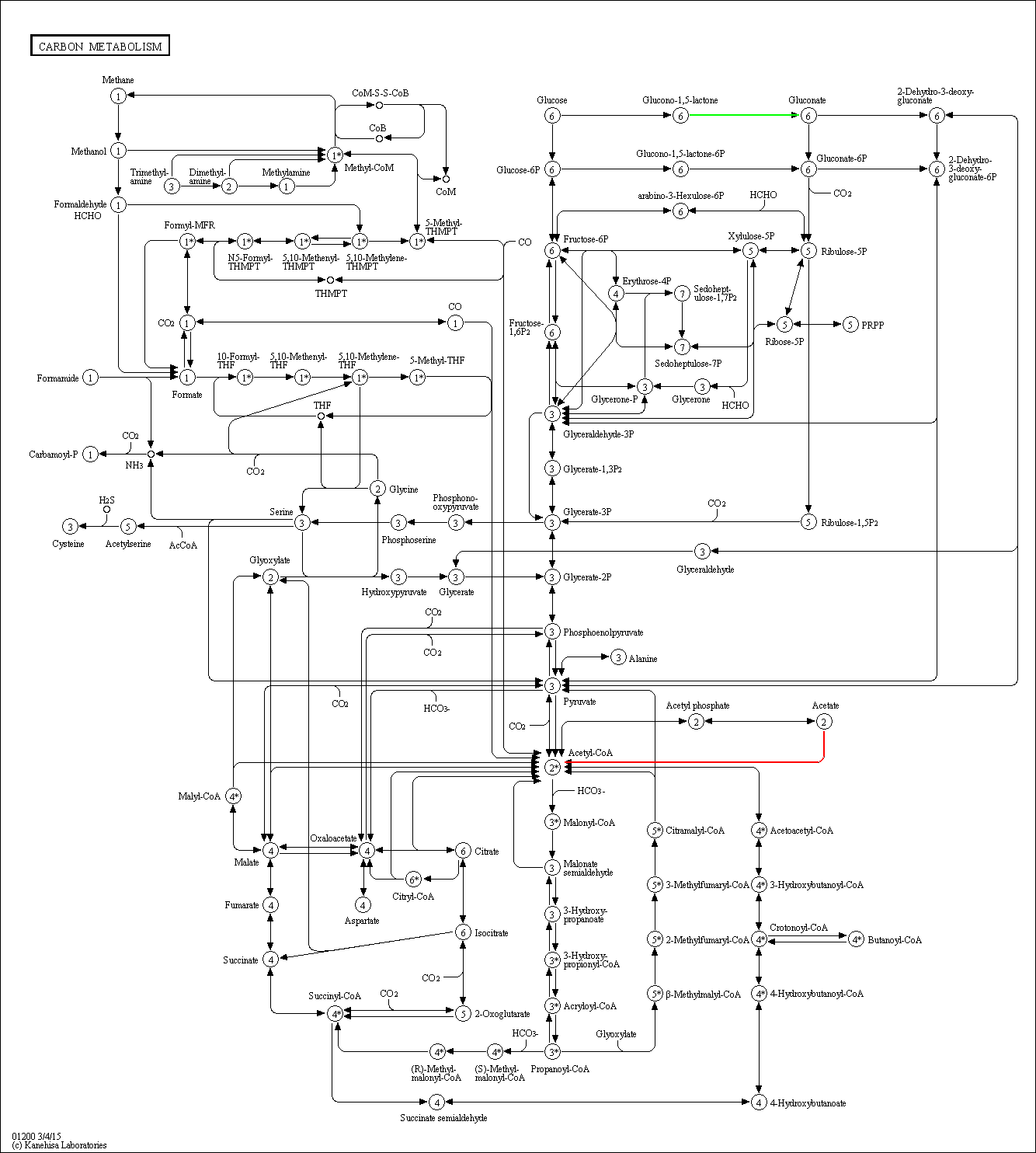

Supplement: S1 File — (ZIP) [file pone.0187105.s011.zip › kegg_map/ko01200.png]

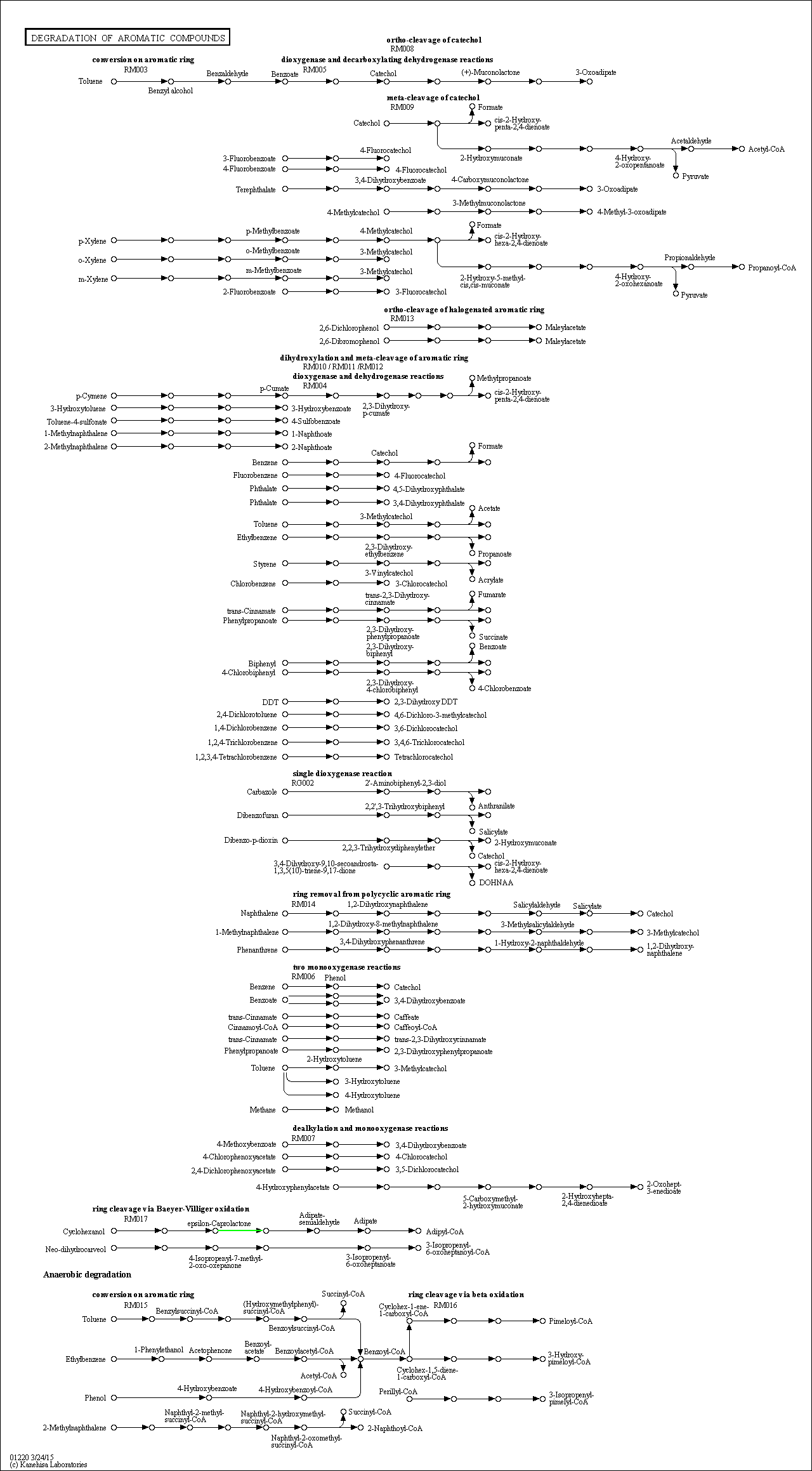

Supplement: S1 File — (ZIP) [file pone.0187105.s011.zip › kegg_map/ko01220.png]

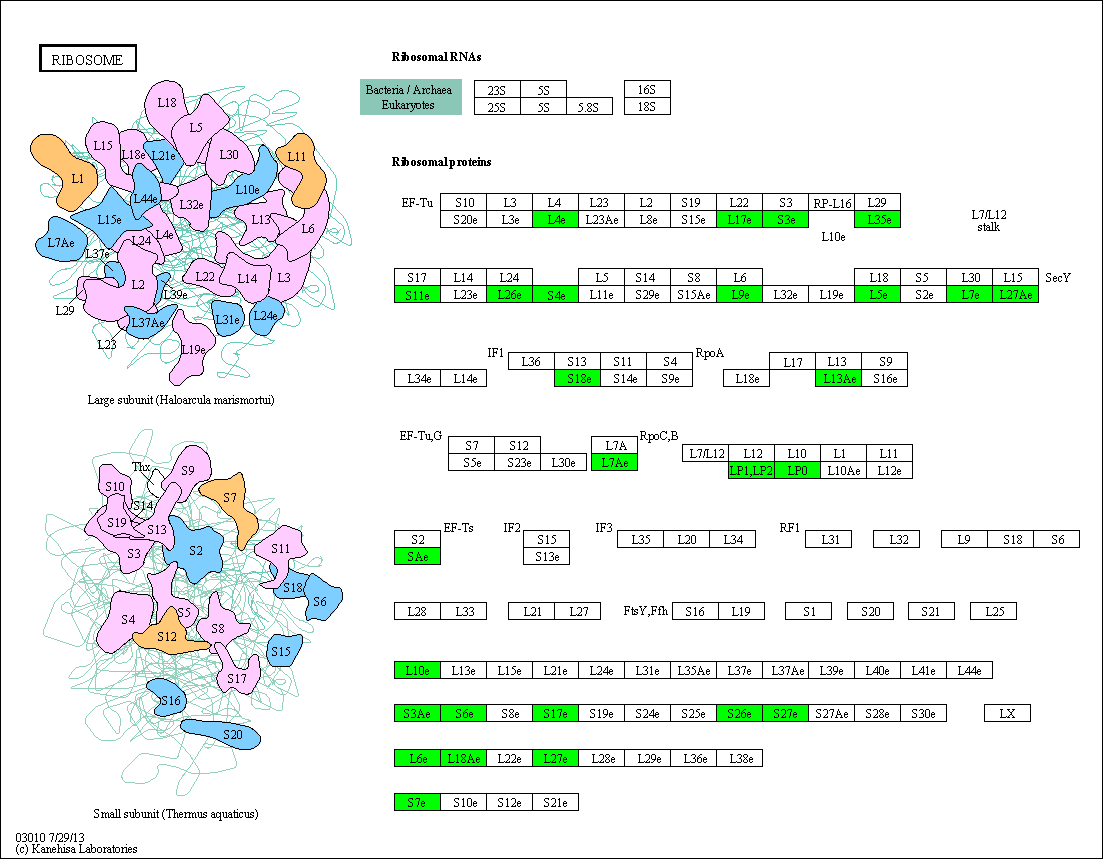

Supplement: S1 File — (ZIP) [file pone.0187105.s011.zip › kegg_map/ko03010.png]

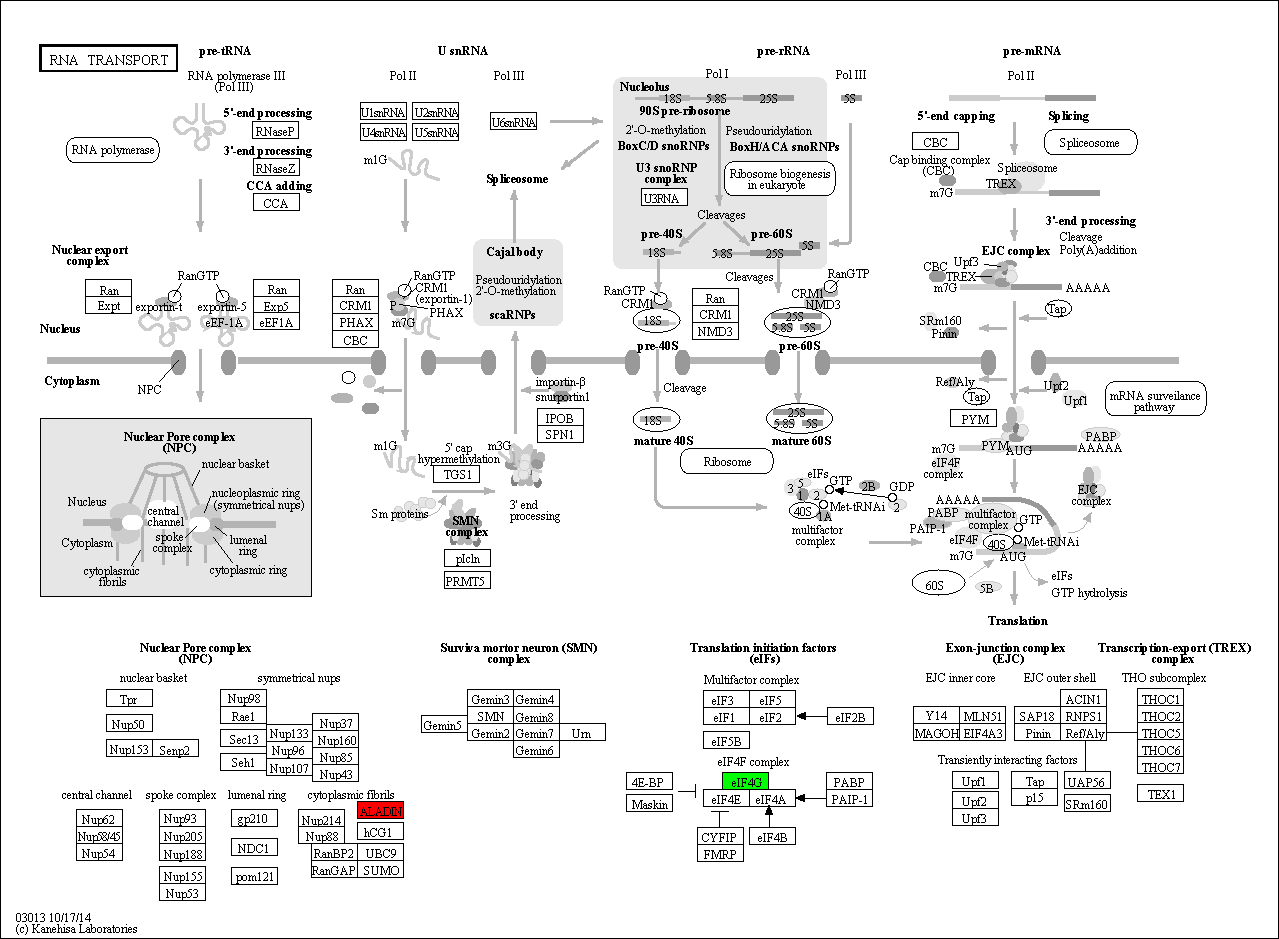

Supplement: S1 File — (ZIP) [file pone.0187105.s011.zip › kegg_map/ko03013.png]

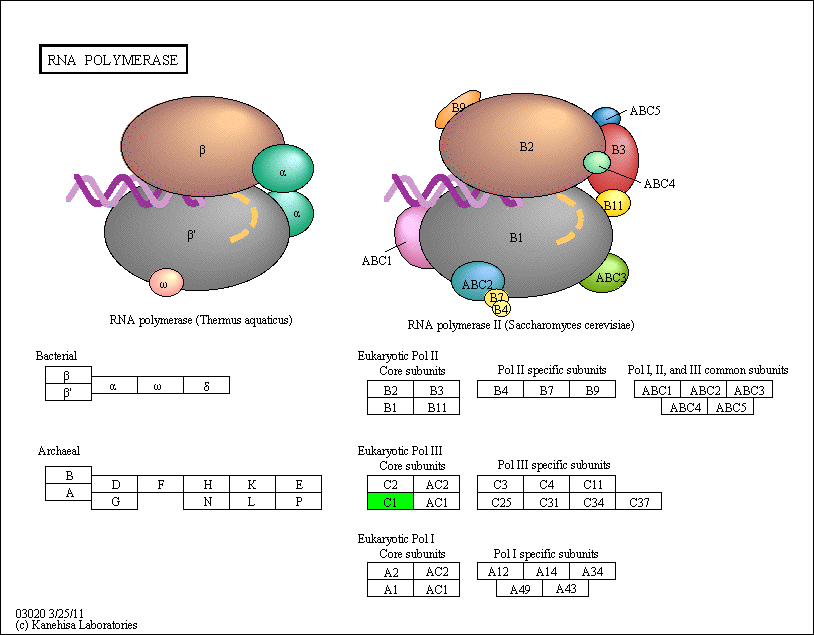

Supplement: S1 File — (ZIP) [file pone.0187105.s011.zip › kegg_map/ko03020.png]

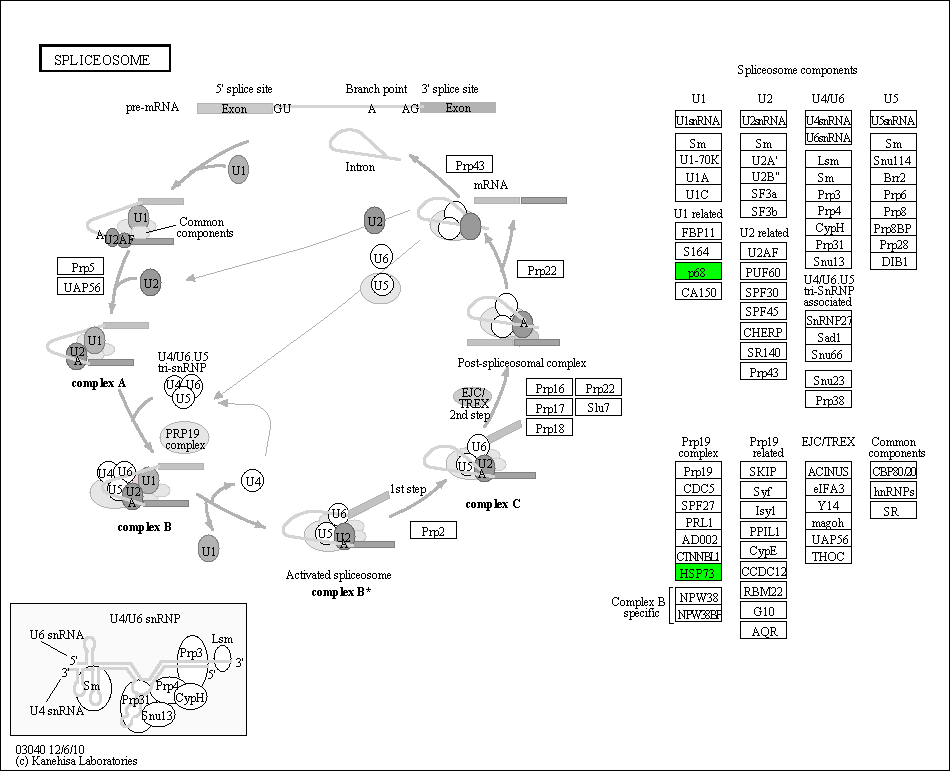

Supplement: S1 File — (ZIP) [file pone.0187105.s011.zip › kegg_map/ko03040.png]

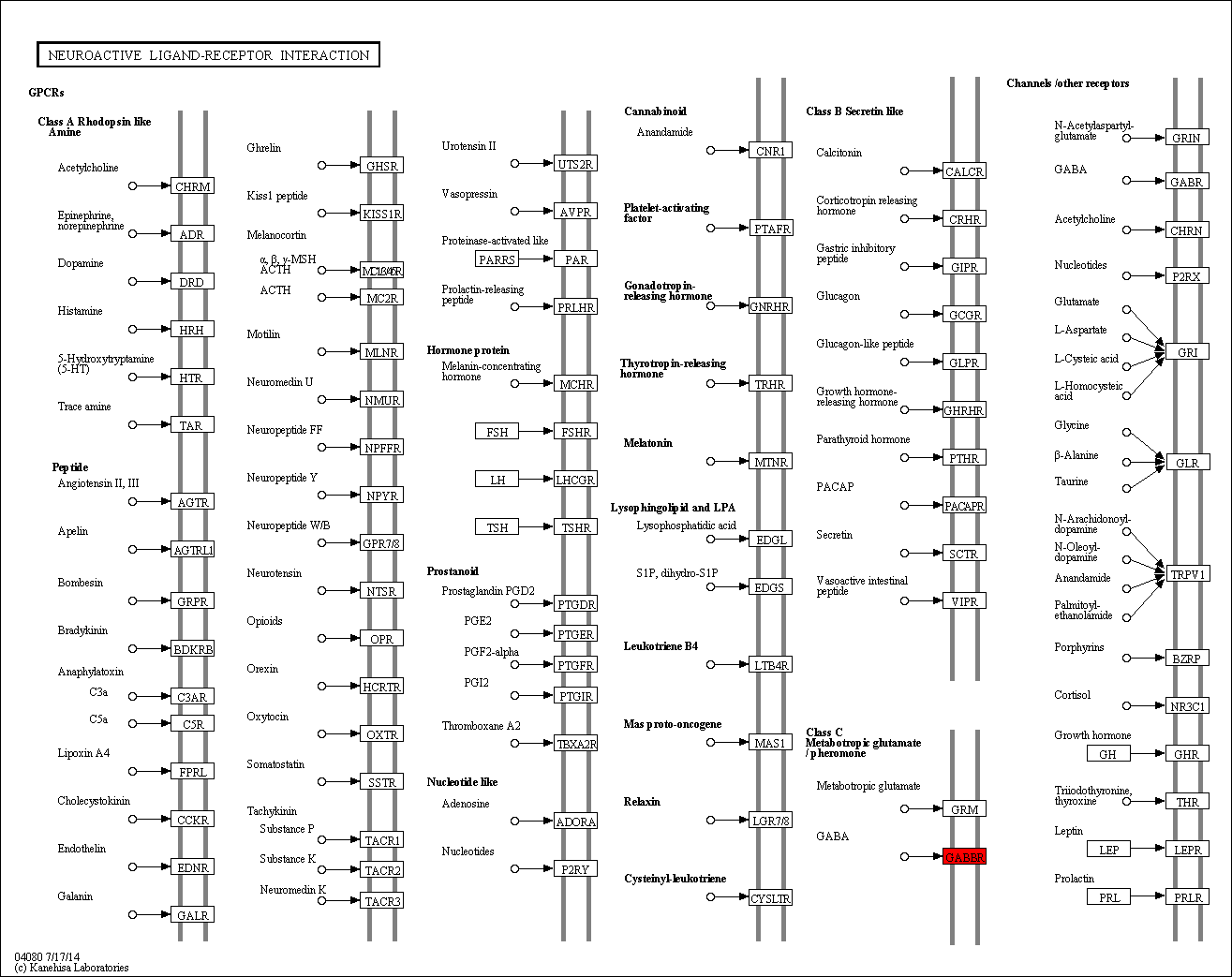

Supplement: S1 File — (ZIP) [file pone.0187105.s011.zip › kegg_map/ko04080.png]

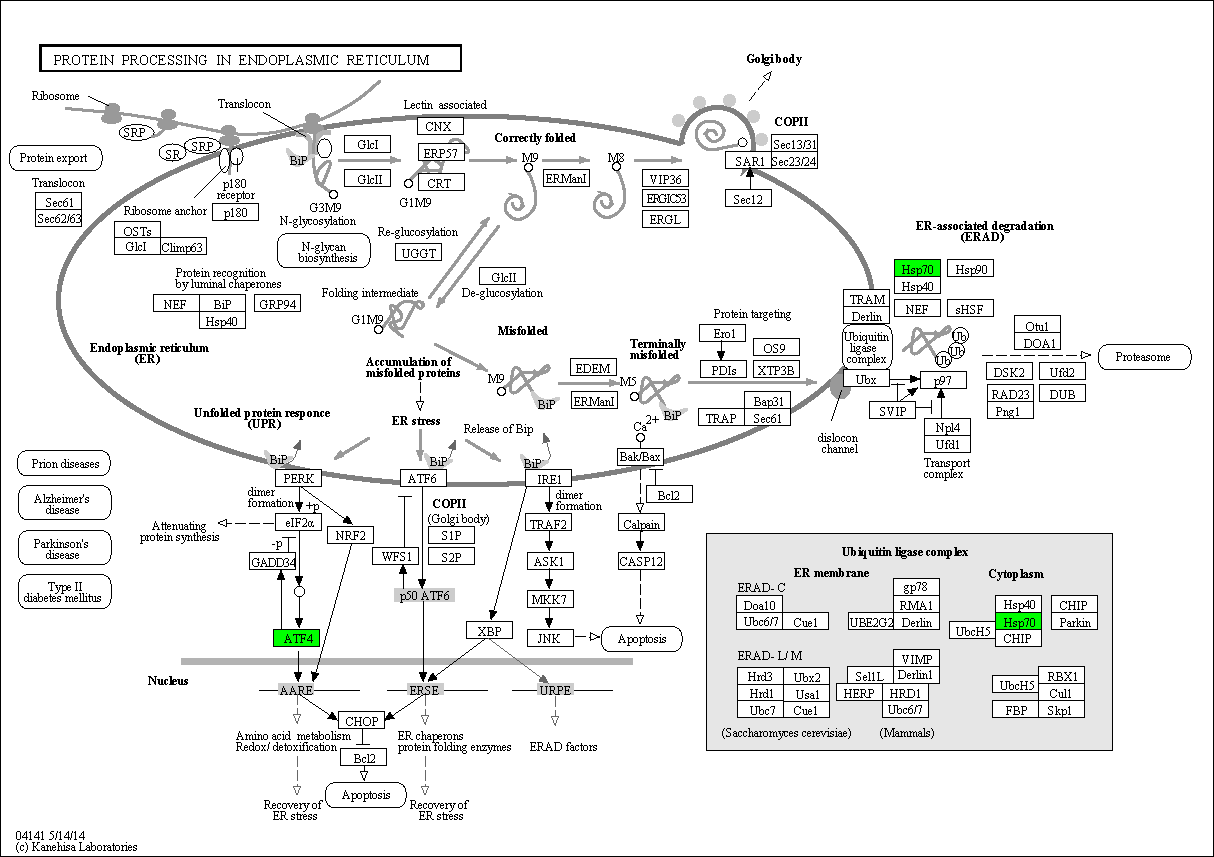

Supplement: S1 File — (ZIP) [file pone.0187105.s011.zip › kegg_map/ko04141.png]

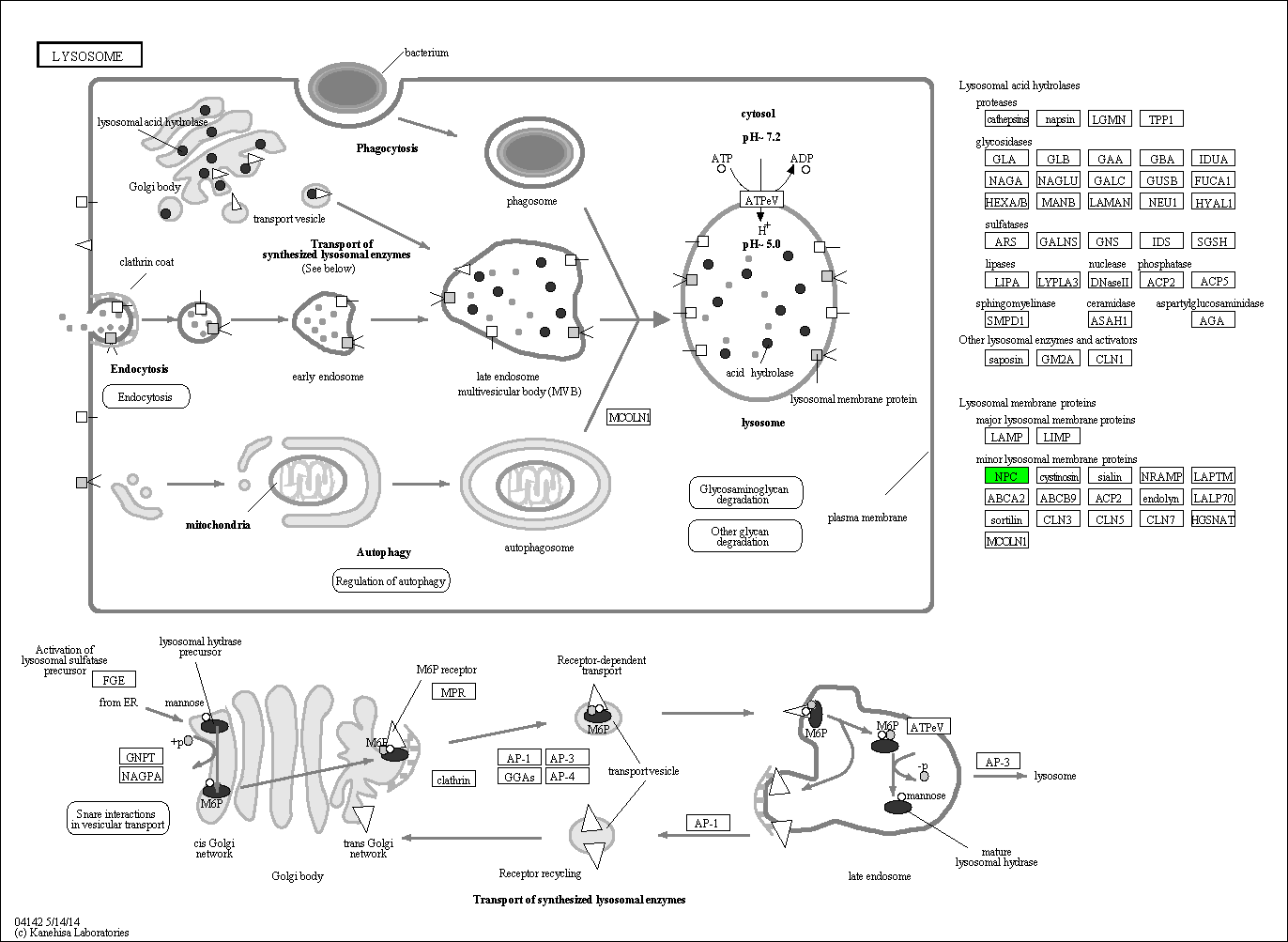

Supplement: S1 File — (ZIP) [file pone.0187105.s011.zip › kegg_map/ko04142.png]

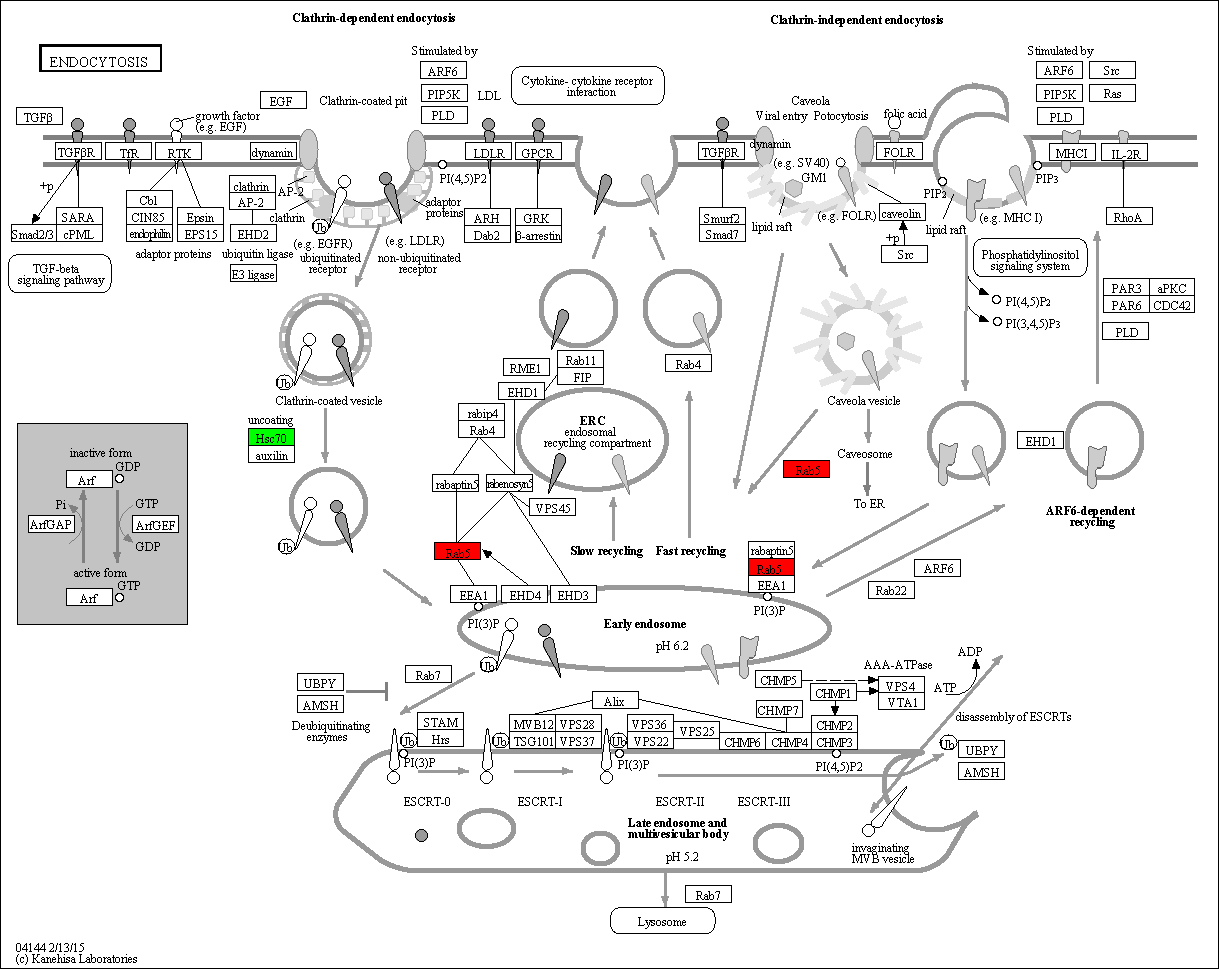

Supplement: S1 File — (ZIP) [file pone.0187105.s011.zip › kegg_map/ko04144.png]

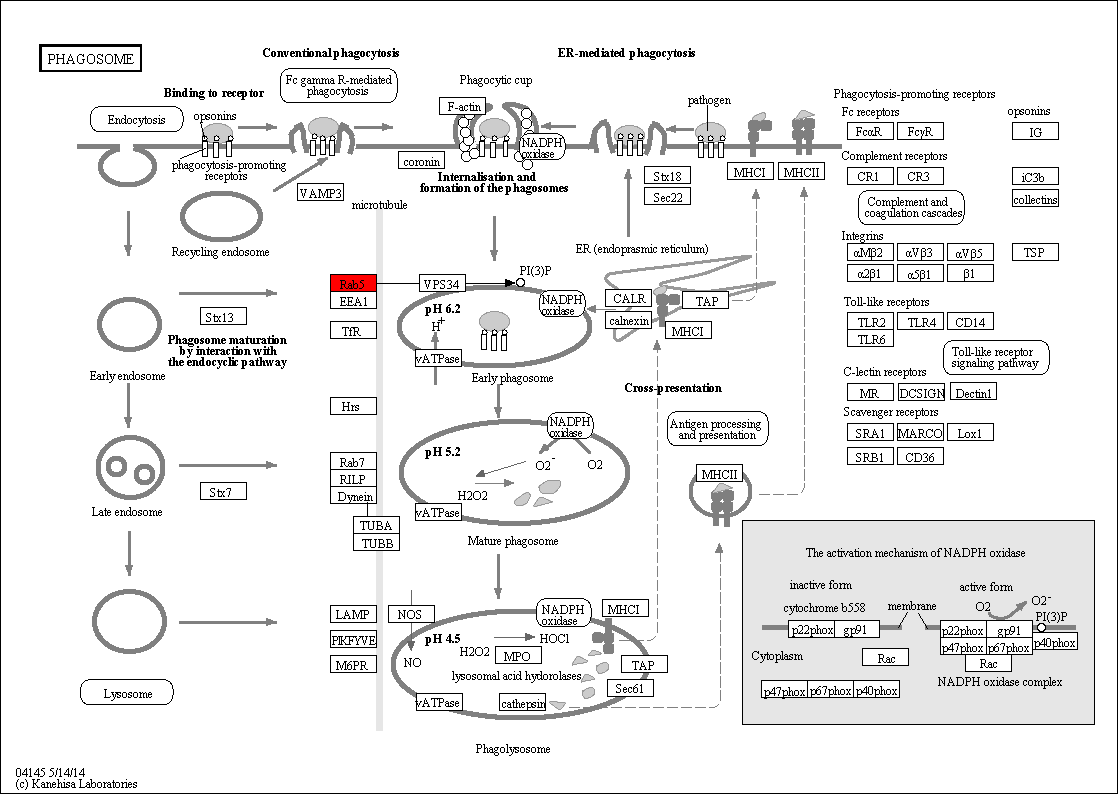

Supplement: S1 File — (ZIP) [file pone.0187105.s011.zip › kegg_map/ko04145.png]

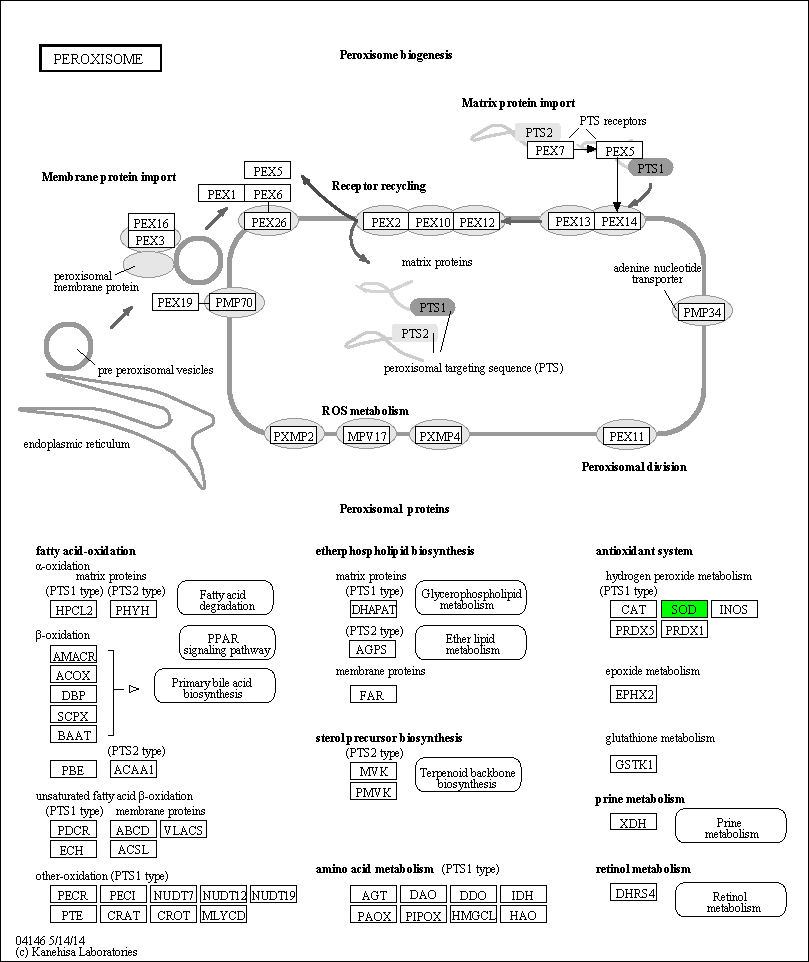

Supplement: S1 File — (ZIP) [file pone.0187105.s011.zip › kegg_map/ko04146.png]

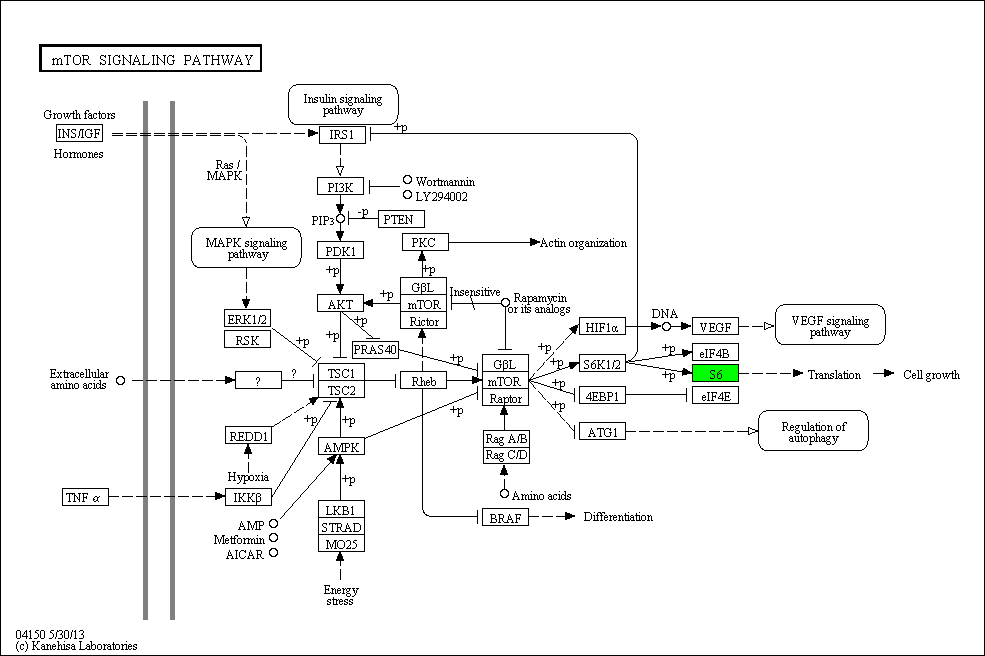

Supplement: S1 File — (ZIP) [file pone.0187105.s011.zip › kegg_map/ko04150.png]

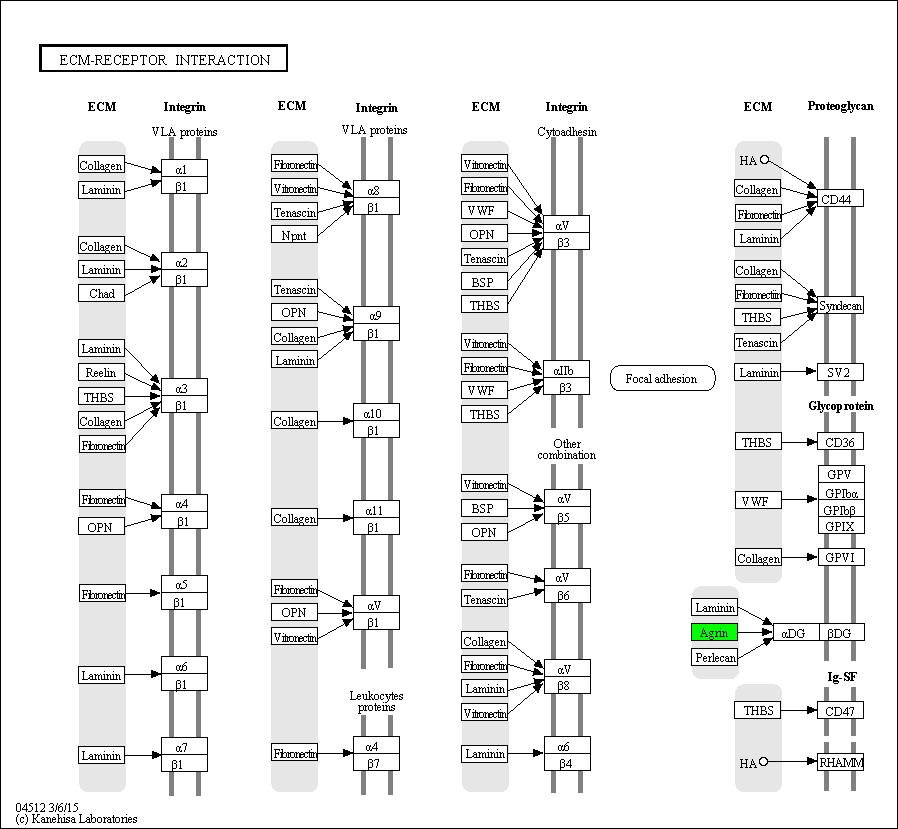

Supplement: S1 File — (ZIP) [file pone.0187105.s011.zip › kegg_map/ko04512.png]
