# Supplementary material for: Differential transcriptome analysis reveals genes related to cold tolerance in seabuckthorn carpenter moth, Eogystia hippophaecolus
Source: PLoS One. 2017 Nov 13;12(11):e0187105. doi: 10.1371/journal.pone.0187105 (PMC5683614; doi:10.1371/journal.pone.0187105)
Supplement: S2 Table — (PDF) [file pone.0187105.s006.pdf]

S2 Table. Primers for real-time PCR

Unigene	Forward primer	Reverse primer
c33229.graph_c0	CGTAGCAGACGGTCGTACTC	CGCAGCTGATTGGTTGAACA
c36418.graph_c0	CGGGGCACAGCTAGCATATT	GGGGGTGTCCATTAATTGCG
c59923.graph_c1	TGCCGCCCTGAAGAAAACAA	ACACATCATGAACCGACGTG
c68914.graph_c0	GTGTAGAGCGTCCCCTGAAT	AGCTGGCTGATGGGTCTAAC
c73192.graph_c0	CGAAGATACAGAGGACCGGC	AGCACCAACTGCTCCCTTAC
c76833.graph_c0	CACAAGCCAGCTACATCCCA	CGAAGCGTGCCTCGTACTAT
c79869.graph_c1	ATCACCTCCTGCCAACGAAG	TCGTCGCTCATTCTCGCTAC
Actin	CGACTTCGAACAGGAGATGG	TCGTCTCATGAATGCCACAG
