# Supplementary material for: Differential transcriptome analysis reveals genes related to cold tolerance in seabuckthorn carpenter moth, Eogystia hippophaecolus
Source: PLoS One. 2017 Nov 13;12(11):e0187105. doi: 10.1371/journal.pone.0187105 (PMC5683614; doi:10.1371/journal.pone.0187105)
Supplement: S1 File — (ZIP) [file pone.0187105.s011.zip › kegg_map/ko00983.html]

ko00983
